# kLDM: Inferring Multiple Metagenomic Association Networks Based on the Variation of Environmental Factors

**DOI:** 10.1016/j.gpb.2020.06.015

**Published:** 2021-02-17

**Authors:** Yuqing Yang, Xin Wang, Kaikun Xie, Congmin Zhu, Ning Chen, Ting Chen

**Affiliations:** 1 Department of Computer Science and Technology and Institute of Artificial Intelligence, Tsinghua University, Beijing 100084, China; 2 Sogou Inc., Beijing 100084, China; 3 Department of Bioinformatics, Key Laboratory of Ministry of Education for Gastrointestinal Cancer, School of Basic Medical Sciences, Fujian Medical University, Fuzhou 350122, China; 4 Peking Union Medical College, Chinese Academy of Medical Science, Beijing 100005, China; 5 Department of Ultrasound, Peking Union Medical College Hospital, Beijing 100005, China; 6 Beijing National Research Center for Information Science and Technology, Beijing 100084, China

**Keywords:** Metagenomics, Association inference, Environmental condition, Bayesian model, Clustering

## Abstract

Identification of significant biological relationships or patterns is central to many metagenomic studies. Methods that estimate association networks have been proposed for this purpose; however, they assume that associations are static, neglecting the fact that relationships in a microbial ecosystem may vary with changes in environmental factors (EFs), which can result in inaccurate estimations. Therefore, in this study, we propose a computational model, called the k-Lognormal-Dirichlet-Multinomial (kLDM) model, which estimates multiple association networks that correspond to specific **environmental conditions**, and simultaneously infers microbe–microbe and EF–microbe associations for each network. The effectiveness of the kLDM model was demonstrated on synthetic data, a colorectal cancer (CRC) dataset, the *Tara* Oceans dataset, and the American Gut Project dataset. The results revealed that the widely-used Spearman’s rank correlation coefficient method performed much worse than the other methods, indicating the importance of separating samples by environmental conditions. Cancer fecal samples were then compared with cancer-free samples, and the estimation achieved by kLDM exhibited fewer associations among microbes but stronger associations between specific bacteria, especially five CRC-associated operational taxonomic units, indicating gut microbe translocation in cancer patients. Some EF-dependent associations were then found within a marine eukaryotic community. Finally, the gut microbial heterogeneity of inflammatory bowel disease patients was detected. These results demonstrate that kLDM can elucidate the complex associations within microbial ecosystems. The kLDM program, R, and Python scripts, together with all experimental datasets, are accessible at https://github.com/tinglab/kLDM.git.

## Introduction

Microbes interact closely with the human body and the environments in which humans live [1], [2], [3]. Metagenomic high-throughput sequencing technology plays a vital role in the study of microbes that inhabit various human body sites and different natural ecological environments. Computational tools have been developed to analyze the microbial constituents of microbiota and the interactions within a microbial community, specifically the interactions among microbes and those between microbes and environmental factors (EFs). EFs are known to be associated with variations of the abundance of microbes, and include the states of human diseases, genotypes of hosts, values of some physiological and biochemical indicators, quantization of people’s lifestyle factors, and concentrations of nutrients [4], [5], [6], [7], [8]. Recently, there has been a rapid increase in large-scale metagenomic studies aiming to discover biological interactions [9], [10], specifically how microbes interact with other microbes and EFs.

**Figure 1:**
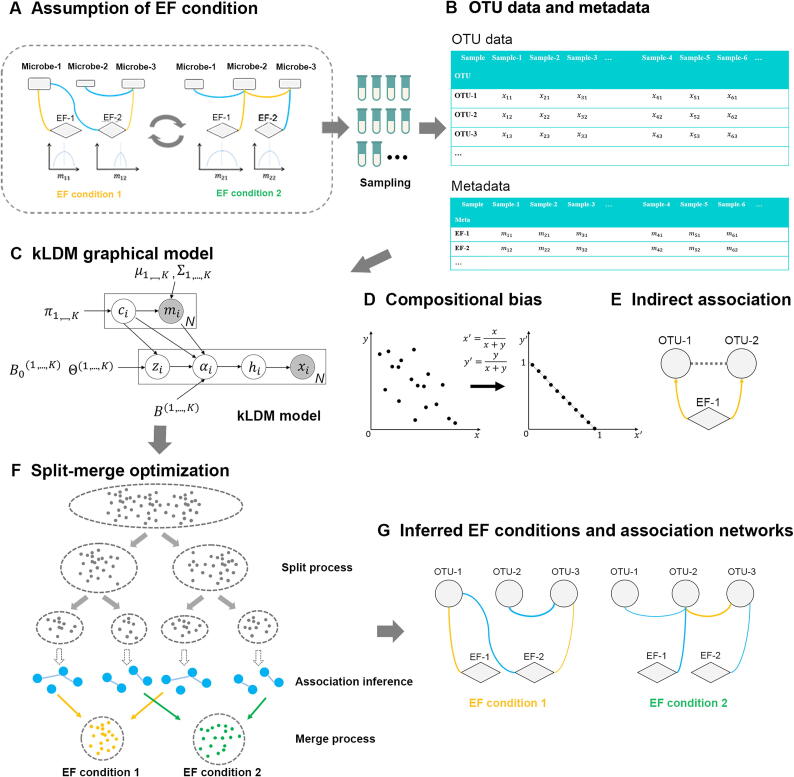
**Schema of the kLDM model** **A.** Multiple environmental conditions are assumed to exist in real environments, and the EF condition can change with time. An EF condition refers to 1) a group of samples in which the EF values fall into a small and defined range, and 2) under this EF condition, interactions within the microbial community are stable. **B.** Sequencing samples with related metadata, possibly from multiple EF conditions, were collected. After data preprocessing, clustering, and annotation, OTU counts were obtained for each sample. The information about which two samples belong to the same EF condition was unknown beforehand. **C.** The kLDM graphical model assumes *K* EF conditions within *N* samples, and infers the number of EF conditions, the associations among OTUs, and the associations between EFs and OTUs under every EF condition. Two matrices, $B^{k}$ and $\Theta^{k}$, respectively record direct EF–OTU associations and OTU–OTU associations for the $k^{\mathrm{th}}$ EF condition. Vectors $x_{i}$ and $m_{i}$ are respectively the OTU counts and values of EFs in the $i^{\mathrm{th}}$ sample. At every EF condition, it is assumed that the values of the EFs follow a multivariate Gaussian distribution, that is, they are parameterized by $\mu_{k}$ and $\Sigma_{k}$. The rest of the variables include the following: $h_{i}$ represents the latent relative ratios of microbes in the $i^{\mathrm{th}}$ sample, $\alpha_{i}$ is the absolute abundance of microbes, $B_{0}^{\left(k\right)}$ represents the impact of unknown factors that affect the abundance of OTUs, $\;c_{i}$ indicates that the $i^{\mathrm{th}}$ sample belongs to the EF condition $c_{i}$, and $\pi_{k}$ is the mixture weight of the $k^{\mathrm{th}}$ EF condition. **D.** Compositional bias caused by the normalization process on the OTU counts. After normalization, the microbial relative abundance sums to one. **E.** The indirect association between OTU-1 and OTU-2 induced by the common EF-1 can be recognized by kLDM, which takes the EF–OTU association into account. **F.** For kLDM, the number of EF conditions and the association networks of every EF condition are estimated by a split-merge optimization algorithm. Both the EF values and associations of microbes are taken into account to determine the EF condition. **G.** Parameters estimated by kLDM can be visualized into EF conditions and association networks. The blue and yellow edges correspond to negative and positive associations, respectively, and the thickness of an edge is proportional to the association value. EF, environmental factor; OTU, operational taxonomic unit; OTU–OTU, microbe–microbe; EF–OTU, environmental factor–microbe.

Biological interactions can be classified as positive or negative relationships among microbes and between EFs and microbes. Commensalism among microbes and microbial dependency on EFs are positive relationships, while parasitism, competition among microbes, and the inhibition of microbes by EFs are negative relationships [11]. Such relationships can be inferred indirectly by predicting the variation patterns of microbial counts and EF values using association inference methods. Furthermore, interactions among microbes and between EFs and microbes depend on the conditions of EFs (named EF conditions), which are defined as some specific ranges of EF values in this study. Under similar EF conditions, interactions in the microbial community are generally stable, but they can change dynamically with the alteration of the EF conditions (Figure 1A). For example, marine microbial interactions may vary by season and depth [12], and interactions among human gut microbes can change according to the onset of diseases and disease states [4], [13]. In these two examples, the environmental conditions of the marine microbial communities are specific seasons and ocean depths, and those of the human gut microbes are particular diseases and disease states. In summary, biological interactions are dynamic, a phenomenon that is considered in the association inference approach utilized in this work.

However, in some studies, environmental conditions are sometimes neither obvious nor known in advance. For example, the American Gut Project [14] collected information on dozens of human lifestyle factors related to individuals’ diets and living habits, as well as metagenomic sequencing data for thousands of individuals. However, it is unclear which subsets of these individuals belong to the same environmental conditions, and therefore which individuals would, by definition, have similar values of some or all of these lifestyle factors, and would also share identical microbial interactions. Besides, even if hosts’ disease states have been obtained, grouping samples into cases and controls while ignoring other EFs may lead to inaccurate analysis results. The underlying patterns of microbial compositions and associations may be very complex and consist of many subgroups, and can be aligned with neither the cases nor the controls. These problems call for novel computational methods with which to discover potential environmental conditions and infer association networks.

In many previous studies, due to the limited numbers of sequencing samples, association inference methods have estimated static association networks using all the samples [15], [16], [17], [18], [19], [20]. These methods can be classified into two categories: methods that compute pairwise associations independently and methods that estimate multiple associations simultaneously. The widely-used Pearson’s correlation coefficient (PCC) and Spearman’s rank correlation coefficient (SCC) methods belong to the former category, as does local similarity association (LSA) [19]. In contrast, CCREPE [16], SparCC [15], SPIEC-EASI [20], CCLasso [18], and mLDM [17] belong to the latter category. The latter methods consider compositional bias [21] caused by the normalization process of microbial read counts, by which microbial read counts are divided by the total sum of read counts to determine the relative abundance. The relative abundance of the $j^{\mathrm{th}}$ operational taxonomic unit (OTU) is defined as $r_{j}=\frac{x_{j}}{\sum_{k}x_{k}}$, where $x_{j}$ is the read count and $\sum_{k\neq j}Cov\left(r_{k},r_{j}\right)=-Var\left(r_{j}\right)$. It is clear that the commonly-proposed normalization process [22] introduces dependency into microbial relative abundance, and, as such, it reduces the efficiency of association inference (Figure 1D). CCREPE, SparCC, and CCLasso estimate OTU–OTU (microbe–microbe) correlations by computing the covariance among microbes, while SPIEC-EASI and mLDM infer conditionally dependent OTU–OTU associations by estimating the precision matrix among microbes. With the exception of mLDM, none of these methods consider associations between EFs and microbes. Taking both the compositional bias and the large variance of read counts into consideration, the method proposed in our previous work, mLDM [17], estimates both OTU–OTU and EF–OTU associations more accurately by removing indirect associations among microbes induced by common EFs (Figure 1E).

However, all the methods noted previously assume only a single biological network, neglecting that biological interactions can be different with the variation of EFs. In this case, if samples from two different environmental conditions are combined to infer associations, the results will reflect the change of environmental conditions rather than real associations under these two environmental conditions; this may lead to false associations and conclusions. For example, association networks of gut microbes in patients with liver cirrhosis and healthy individuals are distinctly different [23], and to predict gut microbial interactions of disease patients, samples from the healthy population should be excluded.

To estimate OTU–OTU associations and EF–OTU associations conditional on environmental conditions, a new hierarchical Bayesian model, the k-Lognormal-Dirichlet-Multinomial (kLDM) model, is proposed in this work (Figure 1). kLDM can automatically determine the number of EF conditions and simultaneously infer complex associations. kLDM considers the compositional bias and large variance of read counts under every EF condition, and estimates both conditionally dependent OTU–OTU associations and direct EF–OTU associations. Most significantly, associations that vary according to environmental conditions can be elaborated by kLDM. It should be noted that the direct or conditionally dependent associations are mathematical concepts that approximate biological interactions, rather than indicate direct biological interactions or causal relationships. In addition, considering that the sequencing of marker genes, such as *16S rRNA / 18S rRNA* genes, is widely used to investigate microbial compositions of samples, kLDM is designed for data produced by such marker gene sequencing technology. To the best of our knowledge, kLDM is the first method that infers multiple association networks based on the variation of EF values. The efficiency and robustness of kLDM are first validated on synthetic datasets via comparing with state-of-the-art approaches. It is then applied to a colorectal cancer (CRC) dataset to demonstrate its ability to find well-defined EF conditions. Its applications on the *Tara* Oceans dataset and the American Gut Project dataset are also explored to discover potential EF conditions and novel biological relationships.

## Method

### Materials used for kLDM evaluation

#### Gut microbial samples from a CRC study

The OTU table and metadata of metagenomic gut *16S rRNA* sequencing data of healthy individuals and patients with CRC were obtained from Baxter et al. [13]. A dataset was constructed, which consisted of 5 known CRC-associated OTUs (*Peptostreptococcus*, *Parvimonas*, *Fusobacterium*, *Porphyromonas*, and *Prevotella*), 112 common OTUs observed in more than half of all 490 samples, and 4 EFs [including the fecal immunochemical test (FIT) result, age, gender, and the diagnostic state of each donor]. The “FIT” result referred to the positive or negative result of FIT. The recorded diagnostic states included “Normal”, “High-risk Normal”, “Adenoma”, “Advanced Adenoma”, and “Cancer”, all of which were determined by colonoscopy examination and the review of biopsies. The diagnostic states were modeled as five numerical values from 1 to 5, with a higher value representing a more serious disease state.

#### Tara Oceans eukaryotic dataset

In the study of the *Tara* Oceans project [24], read counts of marine eukaryotic OTUs were obtained by sequencing the V9 region of *18S rRNA* genes. This project also included 91 genus-level matched eukaryotic symbiotic interactions. After filtering out the OTUs that existed in < 40% of samples and removing samples with missing EFs or abnormal counts, a subset of 221 samples with 67 OTUs related to genus-level symbiotic interactions and 17 EFs was obtained. The 17 EFs included the depth of water, chlorophyll maximum, maximum Brünt-Väisälä frequency, maximum dissolved oxygen, minimum dissolved oxygen, salinity concentration, oxygen concentration, phosphate concentration, silica concentration, chlorophyll concentration, temperature, sunshine duration, moon phase, maximum Lyapunov exponent, residence time, latitude, and longitude.

#### 16S rRNA sequencing samples from the American Gut Project

The OTU table and metadata from the American Gut Project [14] were downloaded from the FTP site (ftp://ftp.microbio.me/AumericanGut), and 22 metadata regarding individuals’ diets and living habits were selected. Among these metadata, 3 factors were associated with living habits (alcohol, exercise, and smoking frequency), and the remaining 19 factors were related to diets (frequencies of the consumption of fermented plants, frozen desserts, fruit, high-fat red meat, home-cooked meals, meat, eggs, milk cheese, milk substitutes, olive oil, probiotics, red meat, salted snacks, seafood, vegetables, vitamin D supplements, vitamin B supplements, whole grains, and whole eggs). The values of the metadata can be categorized as “Never”, “Rarely (less than once/week)”, “Occasionally (1–2 times/week)”, “Regularly (3–5 times/week)”, or “Daily”. For convenience, these categories were recoded into integers from 1 to 5 according to their frequencies. Samples with large (first 1%) or small (last 2%) numbers of reads, as well as those with evenness < 2, were removed. OTUs that existed in < 20% of samples and with an average size of < 50% were filtered out. Finally, 11,946 samples with 216 OTUs and 22 EFs were obtained. The Python script to process the OTU “.biom” file can be found on Github (https://github.com/tinglab/kLDM.git). For every sample, information on individuals’ disease statuses was also recorded, and included cardiovascular disease, small intestinal bacterial overgrowth, mental illness, lactose intolerance, diabetes, inflammatory bowel disease (IBD), irritable bowel syndrome, *Clostridium difficile* infection, cancer, and obesity.

### Assumption of the kLDM hierarchical Bayesian model

The kLDM model assumes that interactions among microbes are regulated by EFs and tend to be constant when environmental conditions do not change, but may vary due to changes of environmental conditions (Figure 1A). In other words, kLDM accounts for variation in the values of EFs. Under the same environmental condition, EF values may fluctuate within a small range, core microbes stay the same, and their associations remain stable. However, for different environmental conditions, EF values, microbial species, and their associations can be quite different. In addition, the distribution of environmental conditions may be continuous and complex [25]. Thus, kLDM uses mixtures of multiple clusters with known distributions to approximate and capture patterns of environmental conditions, with each cluster representing one environmental condition.

### The hierarchical structure of the kLDM model

Let $X={\left\{x_{i}\right\}}_{i=1}^{N}$ be N sequencing samples, where $x_{i}\in N^{P}$ is the $i^{\mathrm{th}}$ sequencing sample with P microbes, and $x_{\mathrm{ij}}$ corresponds to the read count of the $j^{\mathrm{th}}$ microbe or OTU. Values of EFs are represented as $\;M={\left\{m_{i}\right\}}_{i=1}^{N}$, where $m_{i}\in R^{Q}$ is a Q-dimensional vector and $m_{\mathrm{ij}}$ records the value of EF j of the $i^{\mathrm{th}}$ sample. kLDM models the EF vector $m_{i}$ as a multivariate Gaussian distribution, and assumes that the EFs and the entire dataset consist of *K* mixtures of Gaussian components. Thus, samples in which EF vectors belong to the same Gaussian component can be considered as drawn from the same environmental condition and to share identical OTU–OTU and EF–OTU association networks. *K* EF conditions are denoted as $C={\left\{B^{\left(k\right)},\Theta^{\left(k\right)},B_{0}^{\left(k\right)},\mu^{\left(k\right)},\Sigma^{\left(k\right)}\right\}}_{k=1}^{K}$, where matrices $B^{\left(k\right)}$ and $\Theta^{\left(k\right)}$ are the associations between EFs and OTUs and among OTUs for the $k^{\mathrm{th}}$ environmental condition, respectively. $B_{0}^{\left(k\right)}\in R^{Q}$ is a basis vector, and $\mu^{\left(k\right)}$ and $\Sigma^{\left(k\right)}$ are the mean vector and covariance matrix of EFs for the $k^{\mathrm{th}}$ EF condition, respectively. In addition, the weights of *K* EF conditions are *π*$={\left\{\pi_{i}\right\}}_{i=1}^{K}$, where $\pi_{k}$ is the weight of the $k^{\mathrm{th}}$ EF condition, and $\sum_{k=1}^{K}\pi_{k}=1$.

Figure 1C presents the structure of the kLDM model, in which the vectors $x_{i}$ and $m_{i}$ represent the microbial read counts and the EF values of the $i^{\mathrm{th}}$ sample, respectively. The vector $h_{i}$ is a *P*-dimensional latent variable, the value $h_{\mathrm{ij}}$ of which is the latent relative abundance of the $j^{\mathrm{th}}$ microbe in the $i^{\mathrm{th}}$ sample, and $\alpha_{i}$ can be regarded as the absolute abundance vector corresponding to $h_{i}$. kLDM assumes that the absolute abundance $\alpha_{i}$ determines the relative abundance $h_{i}$ of *P* microbes during the sample preparation process, and that the microbial read count vector $x_{i}$ is generated based on the relative microbial abundance during the sequencing process. Sample i can be considered as being collected from a specific EF condition denoted by $c_{i}$ with the mixture weight $\pi_{c_{i}}$. The absolute abundance $\alpha_{i}$ is decided by two sources of associations: 1) associations between EFs and microbes under the $c_{i}^{\mathrm{th}}$ EF condition, which can be parameterized by a linear term $B^{{\left(c_{i}\right)}^{T}}m_{i}$; 2) associations among microbes, the effects of which are included in the EF condition-specific latent variable $z_{i}^{\left(c_{i}\right)}$. The vector $z_{i}^{\left(c_{i}\right)}$ follows a multivariate Gaussian distribution, the parameters of which are determined by the basis vector $B_{0}^{\left(c_{i}\right)}$ and the precision matrix $\Theta^{\left(c_{i}\right)}$, and it records OTU–OTU associations at the EF condition $c_{i}$.

The generative process of the hierarchical Bayesian model, kLDM, is as follows:

$$c_{i}\;\sim \;Categorial\left(\pi \right)$$

$$m_{i}|c_{i}\;\sim \;N\left(\mu_{c_{i}},\Sigma_{c_{i}}\right)$$

$$z_{i}^{\left(c_{i}\right)}|c_{i}\;\sim \;N\left(B_{0}^{\left(c_{i}\right)},\Theta^{{\left(c_{i}\right)}^{-1}}\right)$$

$$\alpha_{i}|c_{i}=exp\left(B^{{\left(c_{i}\right)}^{T}}m_{i}+z_{i}^{\left(c_{i}\right)}\right)$$

$$h_{i}\;\sim \;Dirichlet\left(\alpha_{i}\right)$$

(1) $$x_{i}\;\sim \;Multinomial\left(h_{i}\right)$$

where $B^{\left(c_{i}\right)}$ is a Q×P matrix, in which $B_{\mathrm{qp}}^{\left(c_{i}\right)}$ is the association between the $q^{\mathrm{th}}$ EF and the $p^{\mathrm{th}}$ microbe, and $\Theta^{\left(c_{i}\right)}\in R^{P\times P}$ is a P×P inverse covariance matrix. During the sequencing process, millions of DNA molecules are randomly selected from the DNA library; therefore, the sequencing count $x_{i}$ has a multinomial distribution:

(2) $$P\left(x_{i}|h_{i}\right)=\left(\begin{array}{c} \sum_{j=1}^{P}x_{\mathrm{ij}}\\ x_{i1},\cdots ,x_{\mathrm{iP}} \end{array}\right)\sum_{j=1}^{P}{h_{\mathrm{ij}}}^{x_{\mathrm{ij}}}$$

where the relative abundance $h_{i}$ represents the microbial relative ratios within the DNA library and $\sum_{j=1}^{P}h_{\mathrm{ij}}=1$. Considering the conjugacy of Dirichlet and multinomial distributions, $h_{i}$ follows the Dirichlet distribution as follows:

(3) $$P\left(h_{i}|\alpha_{i}\right)=\frac{\Gamma (\sum_{j=1}^{P}\alpha_{\mathrm{ij}})}{\prod_{j=1}^{P}\Gamma \left(\alpha_{\mathrm{ij}}\right)}\prod_{j=1}^{P}{h_{\mathrm{ij}}}^{\alpha_{\mathrm{ij}}-1}$$

where $\alpha_{i}$ is the vector for the absolute microbial abundance in the $i^{\mathrm{th}}$ sample, which, in turn, determines the relative ratios in the library. In a Dirichlet-multinomial distribution, the covariance of two OTU counts $x_{\mathrm{ij}}$ and $x_{\mathrm{ik}}$ in the $i^{\mathrm{th}}$ sample is $Cov\left(x_{\mathrm{ij}},x_{\mathrm{ik}}\right)\propto -\sum_{j=1}^{P}x_{\mathrm{ij}}\frac{\alpha_{\mathrm{ij}}}{\sum_{j=1}^{P}\alpha_{\mathrm{ij}}}\frac{\alpha_{\mathrm{ik}}}{\sum_{j=1}^{P}\alpha_{\mathrm{ij}}}$, which corresponds to a negative bias and is regulated by both the sequencing depth $\sum_{j=1}^{P}x_{\mathrm{ij}}$ and the OTUs’ relative abundance $\frac{\alpha_{\mathrm{ij}}}{\sum_{j=1}^{P}\alpha_{\mathrm{ij}}}$. The Dirichlet-multinomial distribution is integrated into the hierarchical structure of kLDM to model the compositional characteristics of sequencing count data.

Assuming that microbes in the $i^{\mathrm{th}}$ sample come from EF condition $c_{i}$, their absolute abundances $\alpha_{i}$ are affected by both the EFs $m_{i}$ and interactions within the community. This is modeled into a lognormal distribution that is suitable for most microbial abundances [26], [27]:

(4) $$P\left(\alpha_{i}|c_{i},B^{\left(c_{i}\right)},\Theta^{\left(c_{i}\right)},B_{0}^{\left(c_{i}\right)}\right)=\frac{1}{{\left(2\pi \right)}^{\frac{P}{2}}\Theta^{\frac{1}{2}}}\times exp\left(-\frac{1}{2}{\left(ln\alpha_{i}-\mu_{i}^{\left(c_{i}\right)}\right)}^{T}\Theta \left(ln\alpha_{i}-\mu_{i}^{\left(c_{i}\right)}\right)\right)\prod_{j=1}^{P}\frac{1}{\alpha_{\mathrm{ij}}}$$

where $\mu_{i}^{\left(c_{i}\right)}={B^{\left(c_{i}\right)}}^{T}m_{i}+B_{0}^{\left(c_{i}\right)}$. This can be simplified into the following equation using the relationship between the lognormal and normal distributions:

(5) $$\alpha_{i}|c_{i}=exp\left({B^{\left(c_{i}\right)}}^{T}m_{i}+z_{i}^{\left(c_{i}\right)}\right)$$

where $z_{i}^{\left(c_{i}\right)}|c_{i}\;\sim \;N\left(B_{0}^{\left(c_{i}\right)},\Theta^{{\left(c_{i}\right)}^{-1}}\right)$.

Under the $c_{i}^{\mathrm{th}}$ EF condition, $m_{i}$ follows the multivariate Gaussian distribution $P\left(m_{i}|c_{i}\right)=N\left(\mu_{c_{i}},\Sigma_{c_{i}}\right)$, and the weight of the $c_{i}^{\mathrm{th}}$ EF condition is set to the categorical distribution with the parameter π as follows:

(6) $$P\left(c_{i}=k\right)=\prod_{c=1}^{K}\pi_{c}^{I(c_{i}=k)}$$

Of interest are the parameters $B^{\left(k\right)}$ and $\Theta^{\left(k\right)}$ (k=1,⋯,K), where $B_{\mathrm{ji}}^{\left(k\right)}$ infers direct association between the $i^{\mathrm{th}}$ microbe and $j^{\mathrm{th}}$ EF at the $k^{\mathrm{th}}$ EF condition, and $-\frac{\Theta_{\mathrm{ij}}^{\left(k\right)}}{\sqrt{\Theta_{\mathrm{ii}}^{\left(k\right)}\Theta_{\mathrm{jj}}^{\left(k\right)}}}$ is the conditionally dependent association between the $i^{\mathrm{th}}$ and $j^{\mathrm{th}}$ microbes (Figure 1G).

### Sparse association inference of kLDM in theory

The generative model can be solved theoretically via the expectation–maximization (EM) algorithm and maximum a posteriori (MAP) estimation for the latent variable $z_{i}^{\left(c_{i}\right)}$. It is assumed that the microbe–microbe and EF–microbe associations are sparse and can be inferred by kLDM with sparsity constraints. Detailed equations of inference and optimization can be found in File S1.

However, two potential problems confront this theoretical sparse association inference, thereby limiting the practicality of the model. First, results are very sensitive to the initialization of parameters because the EM algorithm can converge to a local minimum. Second, estimating the number of EF conditions is very time-consuming. Therefore, more effective approaches were explored and an efficient split-merge algorithm was ultimately adopted, as subsequently detailed.

### Implementation of a split-merge algorithm for kLDM

kLDM adopts a split-merge algorithm to estimate the number of EF conditions and sparse OTU–OTU and EF–OTU associations under each EF condition [28]. First, samples are partitioned into fine-grained clusters using the values of EFs, such that samples within a cluster can be regarded as belonging to the same environmental condition. Second, because this partition is not perfect, multiple clusters are merged into one if they share similar environmental conditions and association networks. The final output is a set of sample groups, each with distinct predicted association networks.

More specifically, the split process starts from a single cluster with all samples, and then iteratively selects a cluster and partitions it into two clusters until the number of samples in each cluster is smaller than a pre-defined threshold $N_{\min}$. This process results in the construction of a binary tree for EFs, each node of which corresponds to one cluster of the samples, and each leaf node of which corresponds to a set of samples with similar values of EFs. It is assumed that the EF vector $m_{i}$ follows the multivariate Gaussian distribution, and when a cluster is split in two, two Gaussian mixtures are used to model the distribution of the EFs of the cluster as follows:

(7) $$P\left(m_{i}\right)=\pi_{1}N\left(m_{i}|\mu_{1},\Sigma_{1}\right)+\pi_{2}N\left(m_{i}|\mu_{2},\Sigma_{2}\right)$$

where $\pi_{1}+\pi_{2}=1$ and $\pi_{j}\left(j=1,2\right)$ is the weight of the $j^{\mathrm{th}}$ component. After estimating these parameters via the EM algorithm, each sample of the original cluster is re-assigned to one of the two new clusters with the larger posterior probability.

Association networks are then estimated for the leaves via mLDM [17], which is similar to kLDM when the number of EF conditions is set to one (K=1). The mLDM algorithm was re-implemented with C++ and OpenMP to improve its stability and efficiency, and a comparison of the running time and memory usage by kLDM and mLDM on a single association network inference is presented in Table S1. Inferred associations were used for the merge process.

The merge process aims to recover clusters that are partitioned into multiple leaf nodes as a result of the greedy approach of the split process. The merge process adopts a bottom-to-top strategy, starting from internal nodes at the lowest level and traversing up to the root, to identify leaf nodes for merging. For each internal node, the algorithm traverses down to its left and right branches to search for leaf nodes. The two leaf nodes with the smallest inter-cluster distance, as measured by the Euclidean distance between the mean values of EFs, are merged. kLDM estimates the associations and the extended Bayesian information criterion (EBIC) score for the merged cluster; if its EBIC score is less than the sum of the EBIC scores of the two leaves, then the merged cluster is kept and substitutes the one that has closer mean values of EFs, while the other is discarded. Otherwise, the merged cluster is discarded. This split-merge process reduces the time complexity by limiting the operation at each step to partition one large cluster into two, or to merge two nearby clusters into one. The algorithm can run in parallel, making kLDM very efficient in practice.

### Synthetic data generation process

Synthetic datasets were generated by specifying the numbers of microbes, EFs, and clusters, as well as the range of the number of samples per cluster. Samples were then separately constructed for every cluster via a generative process of the kLDM model. For the $i^{\mathrm{th}}$ cluster (i=1,2,⋯,K), the EF–OTU association matrix $B_{i}$ was produced by sampling uniformly from the interval [-0.5,0.5], with only 15% of the elements set to non-zero. The OTU–OTU association matrix $\Theta_{i}$ was generated using the R package “huge” [29], which outputs a precision matrix for which the adjacency matrix can be random, a cluster, scale-free, a hub, or a band graph. Every graph corresponds to a specific association structure among microbes. Values of the mean vector of the EF of the cluster can be obtained by sampling uniformly from the interval $\left[i,\left(i+0.5\right)\times i\right]$. The Dirichlet-multinomial samples were then produced using the R package “HMP”. For parameters of every setting, ten repetitions were conducted to generate synthetic data, and the mean and standard deviation of the evaluation metrics were then calculated for comparison. The public R-language codes of CCLasso and SPIEC-EASI were used. The implementation of SparCC was provided by SPIEC-EASI. SCC and SCC(all) were implemented directly in the built-in functions of the R language. The “mb” (Meinshausen-Bühlmann) estimation method was set for SPIEC-EASI, and the default parameters of CCLasso, SPIEC-EASI, and SparCC were utilized. In addition, the *P* value was set to 0.05 for SCC and PCC to save the significant associations.

### Evaluation metrics on synthetic data

The receiver operating characteristic (ROC) curves and the area under the curve (AUC) values were utilized to evaluate the performance of association inference. Every cluster estimated by kLDM is represented by two ROC curves, namely the ROC curve of the OTU–OTU associations and that of the EF–OTU associations. When the AUC values were computed, the signs of estimated associations were neglected. Regarding the thresholds for the computation of the AUCs, the absolute values of the calculated associations were selected for kLDM, SparCC, CCLasso, and SPIEC-EASI, and *P* values were used for SCC and PCC. When plotting the ROC curves for SCC and CCLasso, the calculated coefficients were compared with the true correlation matrix, as deduced by the inverse of the precision matrix $\Theta_{i}$. One estimated non-zero association is regarded as a true positive if its value is also non-zero in the ground truth. If the values of the inferred and real results of one association are both zero, the association is a true negative.

## Results

### Comparing kLDM with other methods on synthetic datasets

The performance of kLDM was first assessed using synthetic datasets, and then how the similarity of underlying networks and missing information, *i.e.*, EFs, affects the performance of kLDM was evaluated.

Detailed experiments were conducted to demonstrate the effectiveness of kLDM by comparing it with SCC, CCLasso, and SPIEC-EASI. These three models were included because they were tested in our previous study [17] and exhibited advantages over other methods. SCC estimates both EF–OTU and OTU–OTU associations, CCLasso solves the covariance matrix among microbes, and SPIEC-EASI performs well in inferring the precision matrix among OTUs. For a fair comparison, because none of the methods consider more than one network, data were partitioned into clusters according to the results reported by kLDM, and these programs were applied on each cluster to infer associations. It should be noted that the results of SCC using all samples, denoted as SCC(all), were plotted as the baselines. The ROC curve and AUC value of each cluster were used to compare the performances.

**Figure 2:**
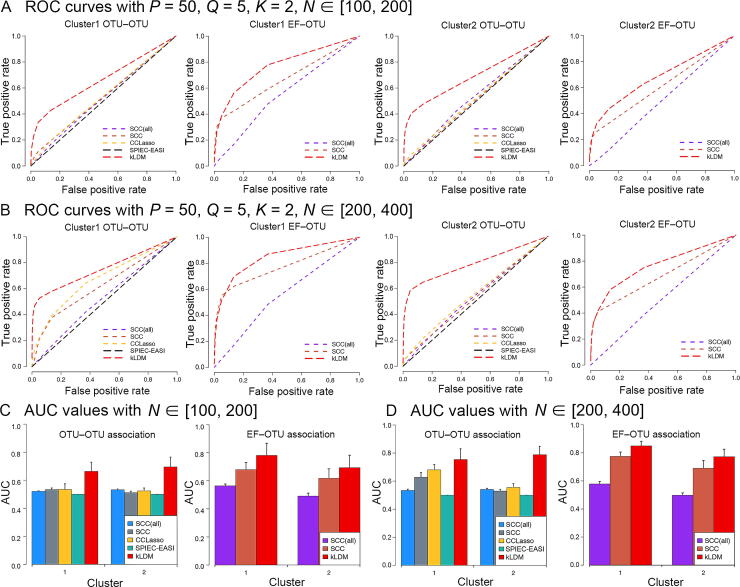
**Comparison of the performance of kLDM with other methods on synthetic data** The performance of kLDM was compared with that of three other association inference methods (SCC, CCLasso, and SPIEC-EASI). *P*, *Q*, *K*, and *N* represent the numbers of OTUs, EFs, EF conditions or clusters, and samples per EF condition, respectively. Values of *P*, *Q*, and *K* were fixed for all panels (*P* = 50, *Q* = 5, *K* = 2). SCC(all) is the result of SCC by assuming that there is only one EF condition in the dataset. **A.** and **C.** Comparisons of ROC curves (A) and AUC values (C) after setting N∈[100,200]. **B.** and **D.** Comparisons of ROC curves (B) and AUC values (D) after setting N∈[200,400]. The ROC curves of the OTU–OTU and EF–OTU associations of two clusters are orderly plotted. The red line corresponds to the result of kLDM. SCC, Spearman’s rank correlation coefficient; ROC, receiver operating characteristic; AUC, area under the curve.

First, the relationship between the number of samples per cluster (*N*) and the efficiency of kLDM was examined by setting the following parameters: *K* = 2 clusters, *P* = 50 microbes, *Q* = 5 EFs, and *N* samples with two ranges separately denoted as $\left[N_{\min},N_{\max}\right]=\left[100,200\right]$ and $\left[N_{\min},N_{\max}\right]=\left[200,400\right]$. These two settings of *N* had identical associations among microbes and between EFs and microbes. As shown in Figure 2, the ROC curves and AUC values of kLDM were consistently better than those of the other methods, and the ability of kLDM to recover OTU–OTU and EF–OTU associations increased with the increase of *N*. However, it was observed that the ROC curves of SCC(all) in most situations were lower, demonstrating the importance of separating samples by environmental conditions. It should be noted that SPIEC-EASI did not efficiently estimate the associations due to its strict model selection. Samples from different EF conditions may introduce many noises and disturb the results of SPIEC-EASI. Additionally, the sensitivities and specificities of the top associations estimated by the five methods were compared using the synthetic dataset with *K* = 2, *P* = 50, *Q* = 5, and *N* ∈ [100,200]. As shown in Table S2, it was evident that kLDM inferred the OTU–OTU and EF–OTU associations with higher sensitivity and superior specificity as compared to the other methods.

**Figure 3:**
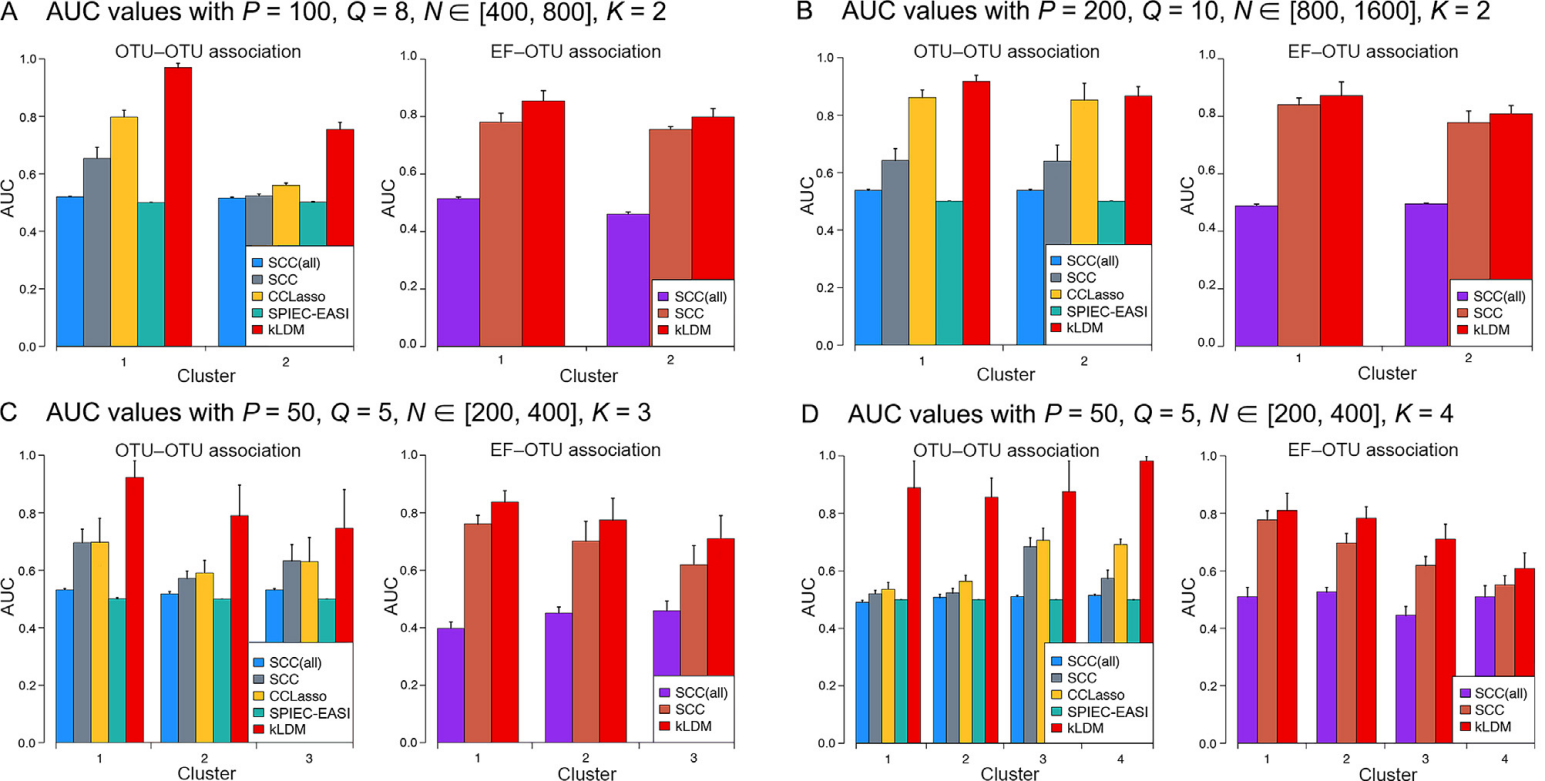
**Evaluation of the scalability of kLDM after increasing the numbers of microbes and EF conditions** **A.** AUC values with 100 OTUs, 8 EFs, and 2 clusters. The number of samples of each cluster ranges from 400 to 800. **B.** AUC values with 200 OTUs, 10 EFs, and 2 clusters. The number of samples of each cluster ranges from 800 to 1600. **C.** AUC values with 50 OTUs, 5 EFs, and 3 clusters. The number of samples of each cluster ranges from 200 to 400. **D.** AUC values with 50 OTUs, 5 EFs, and 4 clusters. The number of samples of each cluster ranges from 200 to 400.

The scalability of kLDM was then investigated with *P* = 100 and *P* = 200, and the results are plotted in Figure 3A and B. In both situations, the AUC values of kLDM were all higher than those of the other approaches, which verifies its fine scalability due to the re-implementation of mLDM with the C++ language and the utilization of parallel computing. Next, kLDM was tested by increasing the number of clusters to 3 and 4 (Figure 3C and D), and kLDM again achieved the best results.

Because the split process of kLDM is affected by the similarity between the EFs of clusters, only the distances between the mean values of EFs of two clusters were changed, and the other parameters were kept the same to examine the performance of kLDM. As is revealed in Table S3, as expected, when the distance between two EF mean vectors was sufficiently large, such as 1.5 or 2.0, which are respectively denoted as “1.5 baseline” and “2.0 baseline”, kLDM accurately inferred the associations. However, the effectiveness of kLDM was found to decrease when the distance became smaller, such as 1.0 (“1.0 baseline”), especially on the inference of OTU–OTU associations. This can be attributed to the tendency of kLDM to group samples together to infer common associations when two clusters have similar values of EFs but different associations.

To test the effect of the similarity of EF–OTU or OTU–OTU associations among clusters on the performance of kLDM, two new datasets were generated for each distance (1.0, 1.5, and 2.0 baseline) by separately setting the value of $B_{i}$ or $\Theta_{i}$ of two clusters (*i* = 1,2) to be equal (“same EF–OTU” or “same OTU–OTU” in Table S3). Compared with the results of the corresponding “baseline”, the AUC values of “same EF–OTU” and “same OTU–OTU” were not significantly changed. From this, it was concluded that the similarity of EFs influences kLDM more than does the similarity of associations among environmental conditions.

In the simulated experiments, all EFs were used to estimate association networks, which may not be feasible in reality because some EFs can be missing. Therefore, new datasets with only partial EFs were simulated to test the effectiveness of kLDM. As shown in Figure S1, the AUC values of kLDM on two clusters are shown with different proportions of EFs retained (20%, 40%, 60%, and 80%) to infer association networks. With the subsets of EFs, the AUC values of kLDM were found to decline. This indicates the importance of including as many EFs as possible. Furthermore, with only 60% of the EFs, kLDM achieved results comparable to those of CCLasso and SCC on the whole dataset, which again demonstrates the superiority of kLDM (Table S4).

### kLDM captures variation in associations of gut microbiota of CRC patients

The relationship between human gut microbiota and CRC has been explored in previous studies [30], [31], [32]. In this work, the metagenomic *16S rRNA* sequencing dataset from Baxter et al. [13] consisting of 117 OTUs and 3 EFs was chosen, and the efficiency of kLDM in capturing the variation of associations in the microbial community was evaluated. kLDM reported two clusters: Cluster 1 was annotated as “Cancer”, and included 90% of “Cancer” samples with significantly higher positive FIT values (*P* = 7.18 × 10^−22^) (Table S5); Cluster 2 was denoted as “Healthy”, and contained 83.7% of healthy samples (“Normal” and “High-risk Normal”) (Table S6). “Adenoma” and “Advanced Adenoma” samples existed in both Cluster 1 and Cluster 2. kLDM did not split samples simply according to the diagnostic state; instead, it took all EF values, microbial abundances, and microbial associations into account.

Different patterns of microbial abundances and associations were found between these two clusters. *Prevotella* was abundant in samples from both clusters, while the other four CRC-associated OTUs were significantly over-represented (*P*  <  0.001) in the samples of Cluster 1 (Figure S2). By comparing the OTU–OTU association networks between Cluster 1 and Cluster 2, as presented in Figure S3, it was found that most gut microbes of “Healthy” samples were connected quite closely and the distribution of associations among microbes was balanced, while few associations were observed between the aforementioned 5 known CRC-associated OTUs and other microbes (Figure S3B). In contrast, associations among the gut microbes of “Cancer” patients were relatively sparse (Figure S3A), and strong correlations were found between specific bacteria (Table 1). *Peptostreptococcus*, *Parvimonas*, *Fusobacterium*, and *Porphyromonas* were found to have strong correlations with each other, but did not connect with other microbes, while *Prevotella* was found to be more associated with other common OTUs, such as *Phascolarctobacterium* and *Clostridium_*XlVa. Based on the differences in microbial abundances and the distributions of associations within the two clusters, the gut microbiota translocation in cancer samples can be found, and *Prevotella* may play a specific role in tumorigenesis.

**Table 1:** **Top associations of CRC-associated microbes for two clusters of the CRC dataset found by kLDM**

**Cluster**	**OTU**	**EF or OTU**	**Association**
Cluster 1 (cancer)	*Parvimonas*	***Porphyromonas***	+0.238
	*Prevotella*	*Phascolarctobacterium*	−0.234
	*Peptostreptococcus*	***Porphyromonas***	+0.220
	*Peptostreptococcus*	***Parvimonas***	+0.191
	*Parvimonas*	***Fusobacterium***	+0.180
	*Porphyromonas*	***Prevotella***	+0.173
	*Peptostreptococcus*	***Fusobacterium***	+0.155
	*Fusobacterium*	***Porphyromonas***	+0.155
	*Porphyromonas*	*Akkermansia*	+0.121
	*Prevotella*	*Clostridium_*XlVa	−0.095
	*Prevotella*	*Clostridium_sensu_stricto*	+0.091
	*Prevotella*	*Clostridium_*IV	−0.090
	*Parvimonas* *Prevotella*	***Prevotella*** *Clostridium_*XlVa	+0.085 −0.082
	*Porphyromonas*	Diagnostic state (EF)	+0.294
	*Fusobacterium*	FIT (EF)	+0.245
	*Peptostreptococcus*	Diagnostic state (EF)	+0.281
Cluster 2 (healthy)	*Prevotella*	Diagnostic state (EF)	+0.274

*Note*: Results of CRC-related OTU–OTU and EF–OTU associations, selected from top 1% weighted associations from the two clusters. Five microbes related to CRC, as reported in previous studies, are labeled in bold and the EFs are underlined. The “Diagnostic state” consists of “Normal”, “High-risk Normal”, “Advanced Adenoma”, “Adenoma”, and “Cancer” states. CRC, colorectal cancer; OTU, operational taxonomic unit; EF, environmental factor; OTU–OTU, microbe-microbe; EF–OTU, environmental factor–microbe; FIT, fecal immunochemical test.

Previous studies have shown that *Peptostreptococcus* and *Fusobacterium* are associated with inflammation [33], [34]. The results of the present study also confirmed that *Peptostreptococcus* was positively correlated with *Fusobacterium* in “Cancer” patients, and that it was over-represented in CRC fecal samples. Table 1 reveals that *Porphyromonas* and *Peptostreptococcus* were found to have positive correlations with diagnostic states in Cluster 1, suggesting the significance of these two bacteria in CRC diagnosis. On the other hand, *Prevotella* was found to be positively associated with the diagnostic states in Cluster 2, indicating a high predictive value, and could be a useful indicator for CRC diagnosis. Research has shown that *Fusobacterium* may act as a passenger microbe to perpetuate tumorigenesis, as a higher load of *Fusobacterium* is related to disease severity [35]. From the results of the present study, patients with cancer that have a positive FIT result are more susceptible to carry *Fusobacterium*.

### Elaborating relationships between marine eukaryote associations and EFs

Next, kLDM was applied on the *Tara* Oceans eukaryotic dataset [24] to explore the associations in natural environments and compare associations predicted by kLDM with known genus-level interactions. kLDM found two clusters corresponding to two EF conditions, namely Cluster 1, consisting of 168 samples with 67 OTUs, and Cluster 2, consisting of 53 samples with 26 OTUs. 41 OTUs in Cluster 2 were filtered out because the number of samples was less than the number of OTUs, and kLDM removed small OTUs to infer associations efficiently. The EFs of the two clusters are listed in Table S7. Cluster 1 had a significantly higher salinity and temperature, but lower oxygen, phosphate, and silica concentrations than Cluster 2. The top 1% associations found in these two clusters, including 23 OTU–OTU and 12 EF–OTU associations in Cluster 1, and 4 OTU–OTU and 5 EF–OTU associations in Cluster 2, were analyzed, and associations with support from the existing literature are listed in Table 2.

**Table 2:** **Top 1% associations of two clusters on the *Tara* Oceans dataset with support from literature**

**Cluster**	**OTU**	**EF or OTU**	**Association**	**Refs.**
Cluster 1	*Amphibelone*	***Phaeocystis***	+0.172	[41]
	*Phaeocystis*	***Amphibelone anomala***	+0.161	[41]
	*Amoebophrya ceratii*	***Cochlodinium_01 fulvescens***	+0.120	[42]
	*Amoebophrya ceratii*	***Cochlodinium_01 fulvescens***	+0.118	[42]
	*Blastodinium mangini*	Salinity (EF)	+0.536	[43]
	*Phaeocystis*	PO_4_ (EF)	+0.508	[44]
	*Amphibelone anomala*	PO_4_ (EF)	+0.439	[45], [46]
Cluster 2	*Blastodinium_06*	Temperature (EF)	+0.549	[43]
	*Blastodinium_06*	Oxygen (EF)	+0.410	[43]
	*Phaeocystis*	Oxygen (EF)	+0.389	[47]

*Note*: Top 1% weighted OTU–OTU and EF–OTU associations are chosen from two clusters, and associations with related literature support are listed in the table. OTUs are labeled in bold and EFs are underlined.

The associations were then matched with known genus-level interactions among the top-*N* associations within each of the two clusters discovered by kLDM. Because known interactions were at the genus-level, an association between two OTUs was regarded to be matched if the OTUs’ genera were identical to two corresponding genera of the known interaction. The results were compared with those when the environmental conditions were not considered and all samples were regarded as one cluster, denoted as “Static”, by limiting kLDM to predict one set of EF–OTU and OTU–OTU association networks. As shown in Table S8, it was clear that the combined predicted associations from the two clusters were similar to the “Static” results, with each cluster consisting of both common and specific OTU–OTU associations.

Different types of known associations detected by kLDM are listed in Table S9. Four known associations, namely “*Phaeocystis*–*Amphibelone*”, “*Vampyrophrya*–*Copepoda*”, “*Amoebophrya*–*Acanthometra*”, and “*Blastodiniaceae*–*Copepoda*”, may be dominant in the ocean because they were inferred from both the whole dataset and the larger cluster (Cluster 1), and the association between *Amoebophrya* and *Protoperidiniaceae* may be strong in a specific EF condition related to Cluster 2. More specifically, the association between OTU-38 and OTU-25, respectively belonging to the genera *Amoebophrya* and *Protoperidiniaceae*, was found to only exist in Cluster 2, in which the mean values of oxygen concentration, phosphate concentration, and silica concentration were higher than those in Cluster 1, and the abundance levels of two OTUs were significantly higher than those in Cluster 1 (*P <* 0.05). The EF condition in Cluster 2 could be more suitable for the growth of the genus *Protoperidiniaceae*, and *Amoebophrya* would then benefit from parasitism with the former. Based on the results from the *Tara* Oceans dataset, the effectiveness of kLDM in elaborating the relationships between OTU–OTU associations and EF values is confirmed.

### Characterizing changes in the association networks of human gut microbes with different lifestyle factors

kLDM has the advantage of analyzing complex datasets with large numbers of samples to infer multiple EF conditions and the corresponding association networks, and this capability was verified on the American Gut Project dataset [14]. kLDM was applied to cluster samples according to lifestyle-related factors and association networks, and two large clusters, Cluster 1 with 6831 samples and Cluster 2 with 5003 samples, and one small cluster, Cluster 3 with 112 samples, were obtained.

Compared with Cluster 1 and Cluster 2, Cluster 3 exhibited different lifestyle patterns and microbiota distributions (Tables S10–S12). Cluster 1 and Cluster 2 together contained almost all disease and healthy people, while Cluster 3 was mainly composed of IBD patients (94.64%), who made up about a quarter of all IBD patients (26.77%). Individuals in Cluster 3 had significantly higher frequencies of alcohol, high-fat red meat, and red meat consumption than those in Cluster 1 and Cluster 2, but they also ate more vegetables and took vitamin D and B supplements and probiotics more frequently. However, their exercise frequency, milk substitute consumption frequency, and milk cheese consumption frequency were distinctly lower (Table S10). For genus-level microbial abundances, the genera *Prevotella*, *Ruminococcus*, and *Sutterella* were more abundant in Cluster 3, but *Bifidobacterium* and *Bacteroides* were less abundant (Table S13). If only IBD samples in each cluster were considered, it can be observed that the IBD samples in Cluster 1 and Cluster 2 were found to be substantially different from those in Cluster 3 based on the aforementioned diet frequencies and genus abundances (Table S14). By comparing the IBD samples in Cluster 1 and Cluster 2, similar lifestyle quantification values and genus abundance levels can be observed, excluding the frequencies of vegetables, fruit, home-cooked meal consumption, and smoking, and the abundance of the genus *Lachnospira*.

The top 1% EF–OTU associations predicted by kLDM were subsequently analyzed (Table S12), and various associations among the three clusters that matched with the findings of published literature are presented. A positive correlation between probiotic frequency and *Bifidobacteria* was found in both Cluster 1 and Cluster 2, which was previously reported by Rajkumar et al. [36]. In people with high animal-protein diets, positive correlations were found between red meat frequency and *Bacteroides* and between poultry frequency and *Ruminococcus* in Cluster 1, and between seafood frequency and *Clostridiales* in Cluster 3, which were all consistent with other research [37]. Associations between high-fat red meat frequency and *Bacteroides* in Cluster 1 and *Clostridiales* in Cluster 2 matched with a rat study that found that the intake of a high-fat diet resulted in disproportionate increases in propionate- and acetate-producing species such as *Clostridiales* and *Bacteroides* [38].

## Discussion

Considering the dynamic nature of microbial interactions, a new hierarchical Bayesian model, kLDM, was proposed to infer associations among microbes and associations between EFs and microbes, under different environmental conditions. Two algorithms, namely a theoretical EM algorithm and a more practical and efficient split-merge algorithm, were then developed to simultaneously estimate both the number of EF conditions and the associations for each EF condition. The effectiveness of kLDM was verified on simulated datasets, as well as the real CRC, *Tara* Oceans, and American Gut Project datasets. Although kLDM was implemented with OpenMP, it can also be accelerated in MPI.

For the synthetic experiment, when the scalability of kLDM was tested (Figure 3A and B), the AUC values of CCLasso were the second-best in most cases, but CCLasso predicted more associations than kLDM, and its ROC curves increased slowly at the beginning and then faster with the increase of the false positive rate. However, the initial high true positive rate is arguably more crucial for scientists wanting to select candidate interactions for validation; in this instance, kLDM presented an advantage. In addition, the influence of the similarity level of EF values between different EF conditions on the performance of kLDM was further explored in detail. The relationship between AUC values and the absolute distance (see the equation in Table S3) between the EF values of two clusters (Figure S4) was plotted based on the “baseline” dataset used in Table S1. When the distance was small (< 2), it was observed that the AUC values of the OTU–OTU and EF–OTU associations increased rapidly with the distance, and when the distance was greater than 2, the power of kLDM tended to be stable. To maintain all AUC values > 0.7, the distance of the EF values of two clusters should be greater than 1.0. The mean values of the inverse Simpson index $n_{\mathrm{eff}}$ of the synthetic datasets were also compared, and the results are exhibited in Table S15. The values of $n_{\mathrm{eff}}$ of the two synthetic datasets with *P* = 100 and *P* = 200 were ≥ 13 and had high compositionality [39]. By considering the results in Table S15 and the AUC values of kLDM in Figure 3, it is clear that kLDM can handle high compositional effects.

For the CRC dataset, the diagnostic state of the donors was included as one EF by kLDM. The diagnostic state was classified according to colonoscopy examination and the review of biopsies, instead of by the microbial compositions and associations; therefore, patients with the same diagnostic state may not have the same underlying microbial associations. In addition, cancer may have different subtypes, and each subtype may have a different association network. Patients who will potentially develop cancer may also have different association networks from those who will not. kLDM was also tested on the CRC dataset using three EFs and excluding the diagnostic state. It was found that the two clusters stayed the same, and the compositions of the diagnostic states in the two clusters did not change. The reason for this may be that the underlying microbial networks of these two clusters were distinct. In addition, kLDM was also compared with a probabilistic clustering model called MicrobeDMM [40], which clusters sequencing samples according only to the microbial composition. From Table S16, it was clear that MicrobeDMM did not distinguish the healthy samples from the CRC samples well, and the gut samples of these two groups were mixed evenly into two clusters. In comparison, in the results of kLDM, most of the “Normal” samples were clustered into one group, while the “Cancer” samples were clustered into the other, which is consistent with the prior knowledge. This demonstrates that using only microbial compositions may not be sufficient, and this result, together with the results of kLDM on the synthetic dataset with partially observable EFs (Figure S1), proves the importance of collecting all EFs.

On the *Tara* Oceans dataset, the top associations found by kLDM were associated with previous studies (Table 2). For Cluster 1, the four listed OTU–OTU associations belonged to two kinds of known microbial interactions. More specifically, the symbiosis between *Phaeocystis* and *Amphibelone anomala*, and the parasitism between *Amoebophrya ceratii* and *Cochlodinium_01 fulvescens*, were captured by kLDM [41], [42]. Associations related to EF values were also found. *Blastodinium mangini* was found to tend to live in seawater with high salinity [43], and the concentration of PO_4_ was found to affect the growth of *Phaeocystis* [44]. In addition, *Amphibelone anomala* has been found to be associated with PO_4_, because it has a close phylogenetic relationship with *Pfiesteria piscicida* [45], which is regulated by phosphate [46]. For Cluster 2, the parasite *Blastodinium_06* was found to be linked to temperature and oxygen, and Skovgaard et al. [43] reported that some *Blastodinium* spp*.* living in warm temperatures can perform photosynthesis. The concentration of oxygen was associated with some *Phaeocystis*, and it has been reported that the bloom of *Phaeocystis globose* causes oxygen depletion [47]. Because most interactions among marine microorganisms are unknown, the explanation in the present study is limited.

For the American Gut Project dataset, kLDM was used to analyze the relationships between lifestyle factors and microbiota, as well as associations within the microbial community. The resultant three clusters exhibited differential frequencies of lifestyle factors, compositions of microbes, and OTU–OTU and EF–OTU associations. Diet preference is among the most influential EFs of the gut microbiome, and it can even determine microbial compositions in the mammalian evolution process [48]. These findings were again reflected in the results of the present study. IBD patients in three clusters were analyzed, and the results indicated the gut microbial heterogeneity of IBD patients; subgroups could therefore potentially be classified by individuals’ lifestyles and microbial communities. The concept of enterotypes in gut microbial communities has been extensively discussed [49], [50], and kLDM may be a useful tool for the discovery of special subgroups of IBD patients.

Whether IBD patients in Cluster 3 differed from other IBD patients in Cluster 1 and Cluster 2 in pathogenesis, clinical characteristics, treatment, and prognosis was also of interest. Notably, although patients in Cluster 3 consumed more probiotics, they still exhibited low abundances of *Bifidobacterium*. Probiotic diets can induce the anti-inflammatory factor IL-10, which improves the gut microenvironment and reduces IBD symptoms [51], [52]. Individuals in Cluster 1 and Cluster 2 had low frequencies of probiotic consumption, which is in agreement with the results of previous studies, but IBD patients from Cluster 3 had high-frequency probiotic consumption (Table S8). Philpott and Girardin [53] reported that IBD patients carrying *NOD2* mutations exhibited decreased transcription of IL-10; thus, it is suspected that patients in Cluster 3 are more likely to carry *NOD2* mutations.

By further comparing the IBD diagnoses among the three clusters (Table S17), all IBD patients in Cluster 3 were found to be those with colonic Crohn’s disease (CD), but such information of IBD samples in the other two clusters is missing. Patients with CD may lack vitamins B and D [54]; therefore, the use of vitamin B and D supplements in Cluster 3 indicates the treatment for such patients. Recently, an “anti-inflammatory” diet, in which high-fat meats are avoided, has been reported to reduce symptoms in patients with IBD [55]. However, the frequency of high-fat red meat consumption was observed for colonic CD patients in Cluster 3, and adjustment in this category may help them in the future. Whether the lifestyles of patients with colonic CD are affected by a doctor’s advice was not included in the dataset, which limits further interpretation.

Taking into account healthy cohorts in Cluster 1 and Cluster 2, it was observed that the genus abundances of the IBD samples in Cluster 1 and Cluster 2 shared more similarity with those of the healthy samples when compared to the IBD samples in Cluster 3 (Table S18). Recently, many microbiota-assisted models have been proposed for the detection of gut lesions, but there have been mixed results [50], [56], [57]. Adding microbial abundance improves sensitivity, but at the cost of specificity. The results of the present study indicate that such decreased specificity may result from the close microbial compositions between some patients and healthy people. Therefore, when microbiota-based prediction models are designed, the heterogeneity within patient samples should be considered.

While it was demonstrated that kLDM is an excellent tool for biologists to interpret microbial associations under multiple environmental conditions, there are some implementation challenges, as well as several possible improvements. For example, the mathematical score (*e.g.*, EBIC score) may not be sufficiently sensitive to separate two environmental conditions. Studies on differential gene co-expression networks may be useful for kLDM. For example, characteristics of nodes in networks, such as the degrees, clustering coefficients, and other mathematical measures that summarize the changes in associations, may be included to develop a more suitable approach to the merging of sub-clusters [58]. In addition, the assumption of Gaussian distributions for the EFs may not be very suitable in the case of categorical data types, and other probabilistic distributions can be considered to model categorical metadata. The scalability of kLDM must also be further expanded so that a large number of rare OTUs with low occurrence can be included in the model. Prior knowledge of microbes and their interactions from known studies may also be very helpful for kLDM to reduce the complexity of association inference and further improve its efficiency [59]. The sample sizes of the real *Tara* Oceans and CRC datasets are relatively small, which is a common phenomenon in current metagenomic studies. However, it is believed that more large-scale datasets like the American Gut Project and Human Microbiome Project datasets will be collected in the future. For these large-scale datasets, kLDM could be the perfect tool for the analysis of complex associations.

## Conclusion

In this work, the kLDM model was proposed to infer EF conditions existing in microbial communities and to predict direct EF–OTU and conditionally dependent OTU–OTU associations under every EF condition while simultaneously considering compositional bias. Compared with traditional methods that estimate static association networks, kLDM has the advantage of illuminating the influences of EFs on associations in microbial communities. The EF condition estimated by kLDM is the result of the comprehensive consideration of EFs, OTU abundances, and associations in the community, which can provide biologists with more insight into the heterogeneity of microbial communities and can identify microbes and interactions regulated by EFs, such as nutrients, lifestyle, and health status. The superiority of kLDM was validated on both synthetic data and real datasets related to the human gut and marine ecosystems. With the deepening of research on the relationship between microorganisms and human diseases, it is expected that kLDM will enable new discoveries of the variations of microbes and OTU–OTU and EF–OTU associations with human health and diets.

## Code availability

The code of kLDM and all experimental datasets are all accessible at https://github.com/tinglab/kLDM.git.

## CRediT author statement

**Yuqing Yang:** Conceptualization, Investigation, Methodology, Software, Writing - original draft. **Xin Wang:** Conceptualization, Investigation, Methodology, Formal analysis, Data curation, Writing - original draft. **Kaikun Xie:** Investigation, Writing - original draft. **Congmin Zhu:** Investigation, Writing - original draft. **Ning Chen:** Conceptualization, Supervision, Writing - review & editing. **Ting Chen:** Conceptualization, Supervision, Writing - review & editing. All authors have read and approved the final manuscript.

## Competing interests

All authors declare no conflicts of interest.

## Supplementary material

Supplementary File S1 **Sparse association inference of kLDM in theory**

Supplementary Figure S1 **AUC scores of kLDM when EFs with different ratios are utilized** The ‘baseline’ dataset in Table S3 was used, and the results when only 20%, 40%, 60%, and 80% of the EFs found in the dataset were compared. EFs were orderly selected and set to zero because they equally contributed to EF-OTU associations in the simulation experiment. The changes of the AUCs of the OTU-OTU and EF-OTU associations of two clusters are shown.

Supplementary Figure S2 **CRC-associated microbial relative abundances of two clusters**

Supplementary Figure S3 **OTU-OTU association networks for cancer (Cluster 1) and healthy (Cluster 2) clusters** Edges represent associations with absolute weight among the top 1%. Red and green edges represent positive and negative associations, respectively. The width of an edge is proportional to the association’s absolute weight. Only microbes with associations in either Cluster 1 or Cluster 2 are shown. The positions of OTUs in A and B are exactly identical, and the sizes of nodes are correlated with their average abundance in all samples. 5 CRC-associated microbes (*Peptostreptococcus, Parvimonas, Fusobacterium, Porphyromonas*, and *Prevotella*) are arranged closely and colored in blue-green, and their associated microbes are labeled in red.

Supplementary Figure S4 **The relationship between the effectiveness of kLDM and the similarity of the EFs of EF conditions** The ‘baseline’ dataset in Table S3 was used again, and the distances between the mean values of the EFs of the two clusters were changed from 0.1 to 10.0. For each distance, AUC scores of the estimated OTU-OTU associations (“Cluster 1 OTU-OUT” and “Cluster 2 OTU-OUT”) and EF-OTU associations (“Cluster 1 EF-OUT” and “Cluster 2 EF-OUT”) of the two clusters were calculated.

Supplementary Table S1 **Comparison of the running time and memory usage of kLDM and mLDM on a single association network inference**

Supplementary Table S2 **Mean values of the sensitivities and specificities of the top N associations of five different methods**

Supplementary Table S3 **AUC scores of kLDM considering the influence of the respective similarities of environmental conditions, EF-OTU associations, and OTU-OTU associations**

Supplementary Table S4 **AUC scores of kLDM when EFs with different ratios are utilized and other methods use the full dataset**

Supplementary Table S5 **Mean values of the metadata of two clusters on colorectal cancer data**

Supplementary Table S6 **Compositions of the diagnostic states in two clusters on colorectal cancer data**

Supplementary Table S7 **Mean values of the EFs of two clusters on the Tara Oceans dataset**

Supplementary Table S8 **Matched genus-level interactions on the Tara Oceans dataset by kLDM and “Static”**

Supplementary Table S9 **Types of matched genus-level interactions in the results of kLDM and “Static”**

Supplementary Table S10 **20 EFs of three clusters predicted by kLDM on the American Gut Project dataset**

Supplementary Table S11 **Health status of people in three clusters on the American Gut Project dataset**

Supplementary Table S12 **OTU abundance, EF-OTU, and OTU-OTU associations of three clusters on the American Gut Project dataset**

Supplementary Table S13 **Genus-level relative abundances of three clusters on the American Gut Project dataset**

Supplementary Table S14 **EF values and genus-level relative abundances of IBD samples in three clusters**

Supplementary Table S15 **Values of the inverse Simpson index of synthetic datasets corresponding to Figure 2 and 3**

Supplementary Table S16 **Compositions of the diagnostic states in two clusters estimated by MicrobeDMM on colorectal cancer data**

Supplementary Table S17 **IBD diagnosis of IBD patients in three clusters on the American Gut Project dataset**

Supplementary Table S18 **EF values and genus-level abundances of healthy and IBD samples in C1 and C2**
