# Supplementary material for: kLDM: Inferring Multiple Metagenomic Association Networks Based on the Variation of Environmental Factors
Source: Genomics Proteomics Bioinformatics. 2021 Feb 17;19(5):834–47. doi: 10.1016/j.gpb.2020.06.015 (PMC9170748; doi:10.1016/j.gpb.2020.06.015)
Supplement: Supplementary File S1 — Sparse association inference of kLDM in theory [file mmc1.docx]

## File S1 Sparse association inference of kLDM in theory

The generative model can be solved theoretically via Expectation Maximization (EM) algorithm and maximum a posteriori (MAP) estimation for the latent variable$z_{i}^{(c_{i})}$. We assume that associations among microbes and between environmental factors and microbes are sparse and can be inferred by kLDM with sparsity constraints. The logarithm of posterior distribution of the latent variables $Z$ is below,

$$\ln P(Z|X,M)\propto\ln P(X,Z,M)=\ln\prod_{i=1}^{N} P(x_{i},z_{i},m_{i})$$

$$\begin{matrix} =\sum_{i=1}^{N} \ln P(x_{i},z_{i},m_{i})=\sum_{i=1}^{N} \ln\sum_{c_{i}=1}^{K} P(x_{i},z_{i}^{(c_{i})},m_{i},c_{i}) \\ =\sum_{i=1}^{N} \ln\sum_{c_{i}=1}^{K} q(c_{i})\frac{P(x_{i},z_{i}^{(c_{i})},m_{i},c_{i})}{q(c_{i})} \end{matrix}$$

$$\geq\sum_{i=1}^{N} \sum_{c_{i}=1}^{K} q(c_{i})\ln P(x_{i},z_{i}^{(c_{i})},m_{i},c_{i})-\sum_{i=1}^{N} \sum_{c_{i}=1}^{K} q(c_{i})\ln q(c_{i})$$

where $\sum_{c_{i}=1}^{K} q(c_{i})=1$ and only when $q(c_{i})=P(c_{i}|x_{i},z_{i}^{(c_{i})},m_{i})$, the inequality holds according to the Jensen’s inequality. Suppose $r_{ik}=q(c_{i}=k)$, then in the E-step, values of$r_{ik}$ for $i=1,...,N$ and $k=1,...,K$ are calculated where

$$\begin{matrix} P(c_{i}=k,x_{i},z_{i}^{(c_{i})},m_{i})= \\ P(x_{i}|\alpha_{i}^{(c_{i})})P(z_{i}^{(c_{i}=k)}|c_{i}=k)P(m_{i}|c_{i}=k)P(c_{i}=k) \end{matrix}$$

$$P(c_{i}=k|x_{i},z_{i}^{(c_{i})},m_{i})=\frac{P(c_{i}=k,x_{i},z_{i}^{(c_{i})},m_{i})}{\sum_{c_{i}=1}^{K} P(c_{i},x_{i},z_{i}^{(c_{i})},m_{i})}=\frac{P_{c_{i}=k}}{\sum_{c_{i}=1}^{K} P_{c_{i}}}$$

$$P_{c_{i}=k}=\frac{T(\alpha_{i}^{(k)}+x_{i})}{T(\alpha_{i}^{(k)})}\frac{|\Theta^{(k)}|^{\frac{1}{2}}}{|\Sigma_{k}|^{\frac{1}{2}}}\pi_{k}\exp\left(-\frac{1}{2}D_{z}^{(k)}-\frac{1}{2}D_{m}^{(k)}\right)$$

with $T(\alpha_{i}^{(k)})=\frac{\prod_{j=1}^{P} \Gamma(\alpha_{ij})}{\Gamma(\sum_{j=1}^{P} \alpha_{ij})}$, $\alpha_{i}^{(c_{i})}=\exp(B^{(c_{i})^{T}}m_{i}+z_{i}^{(c_{i})})$, $D_{z}^{(k)}=(z_{i}^{(k)}-B_{0}^{(k)})^{T}\Theta^{k}(z_{i}^{(k)}-B_{0}^{(k)})$ and $D_{m}^{(k)}=(m_{i}^{(k)}-\mu_{k})^{T}\Sigma_{k}^{-1}(m_{i}^{(k)}-\mu_{k})$.

In the M-step, we minimize following problems:

$$\begin{matrix} \min_{B^{(c_{i})},B_{0}^{(c_{i})},\Theta^{(c_{i})},z_{i}^{(c_{i})},\mu_{c_{i}},\Sigma_{c_{i}},\pi_{c_{i}}}f(B^{(c_{i})},B_{0}^{(c_{i})},\Theta^{(c_{i})},z_{i}^{(c_{i})},\mu_{c_{i}},\Sigma_{c_{i}},\pi_{c_{i}}) \\ +\sum_{c_{i}} \frac{\lambda_{1}}{2}||\Theta^{(c_{i})}||_{1}+\sum_{c_{i}} \lambda_{2}||B^{(c_{i})}||_{1} \end{matrix}$$

where $\begin{matrix} f(B^{(c_{i})},B_{0}^{(c_{i})},\Theta^{(c_{i})},z_{i}^{(c_{i})},\mu_{c_{i}},\Sigma_{c_{i}},\pi_{c_{i}})= \\ -\frac{1}{N}\sum_{i=1}^{N} \sum_{k=1}^{K} r_{ik}\ln P(x_{i},z_{i}^{(c_{i}=k)},m_{i},c_{i}=k)=-\frac{1}{N}\sum_{i=1}^{N} \sum_{k=1}^{K} r_{ik}\left(\ln T(\alpha_{i}^{(k)}+x_{i})-\right. \end{matrix}$

$\ln T(\alpha_{i}^{(k)})+\frac{1}{2}\ln|\Theta^{(k)}|-\frac{1}{2}\ln|\Sigma_{k}|-\frac{1}{2}D_{z}^{(k)}-\frac{1}{2}D_{m}^{(k)}+\ln\pi_{k})$ . It should be noted that $L_{1}-norm$ penalties are added on both $\Theta^{(c_{i})}$ and $B^{(c_{i})}$ with positive parameters$\lambda_{1}$ and $\lambda_{2}$, which can help to resolve overfitting when the number of unknown parameters are much larger than the samples’ size. Then the objective function can be optimized by estimating parameters $B^{(c_{i})},B_{0}^{(c_{i})},\Theta^{(c_{i})},z_{i}^{(c_{i})},\mu_{c_{i}},\Sigma_{c_{i}},\pi_{c_{i}}$alternately.

For $z_{i}^{(k)}$ , we minimize the objective function with respect to $z_{i}^{(k)}$ using the L-BFGS algorithm [1] and the derivative of $z_{i}^{(k)}$is as below,

$$\begin{matrix} \frac{\partial f}{\partial z_{ij}^{(k)}}=-\frac{1}{N}r_{ik}(\tilde{\Gamma}^{'}(\alpha_{ij}^{(k)}+x_{ij})-\tilde{\Gamma}^{'}(\alpha_{ij}^{(k)})+\tilde{\Gamma}^{'}(\sum_{j=1}^{P} \alpha_{ij}^{(k)}) \\ -\tilde{\Gamma}^{'}(\sum_{j=1}^{P} (\alpha_{ij}^{(k)}+x_{ij})))\alpha_{ij}^{(k)}+\frac{1}{N}r_{ik}\Theta_{j:}^{(k)}(z_{i}^{(k)}-B_{0}^{(k)}) \end{matrix}$$

where $\tilde{\Gamma}^{'}(x)$is the digamma function of the variable $x$ and $\Theta_{j:}^{(k)}$ is the $j^{th}$ row of the matrix $\Theta^{(k)}$. For $\Theta^{(k)}$, the objective function is $\min_{\Theta^{(k)}}-\ln\Theta^{(k)}+tr(S^{(k)}\Theta^{(k)})+\lambda_{k}||\Theta^{(k)}||_{1}$ and the inverse covariance matrix can be estimated efficiently by the QUIC method [2] with$S^{(k)}=\sum_{i=1}^{N} \frac{r_{ik}}{\sum_{j=1}^{N} r_{jk}}(z_{i}^{(k)}-B_{0}^{(k)})(z_{i}^{(k)}-B_{0}^{(k)})^{T}$. For $B^{(c_{i})}$, the proximal quasi-Newton approach [3] can minimize the objective function with $L_{1}$ penalty and the derivative of $B_{ij}^{(k)}$is needed as below,

$$\begin{matrix} \frac{\partial f}{\partial B_{ij}^{(k)}}=-\frac{1}{N}\sum_{h=1}^{N} r_{hk}(\tilde{\Gamma}^{'}(\alpha_{hj}^{(k)}+x_{hj})-\tilde{\Gamma}^{'}(\alpha_{hj}^{(k)}) \\ +\tilde{\Gamma}^{'}(\sum_{t=1}^{P} \alpha_{ht}^{(k)})-\tilde{\Gamma}^{'}(\sum_{t=1}^{P} \alpha_{ht}^{(k)}+x_{ht}))\alpha_{hj}^{(k)}m_{hi} \end{matrix}$$

. Then$B_{0}^{(c_{i})}$ is calculated based on $z^{(c_{i})}$with $B_{0}^{(k)}=\sum_{i=1}^{N} r_{ik}z_{i}^{(k)}/\sum_{i=1}^{N} r_{ik}$ . Other variables $\mu_{c_{i}},\Sigma_{c_{i}},\pi_{c_{i}}$can be computed directly as $\mu_{k}=r_{ik}m_{i}/\sum_{i=1}^{N} r_{ik}$ , $\Sigma_{k}=\sum_{i=1}^{N} r_{ik}(m_{i}-\mu_{k})(m_{i}-\mu_{k})^{T}/\sum_{i=1}^{N} r_{ik}$ and $\pi_{k}=\sum_{i=1}^{N} r_{ik}/N$ . In order to decide the number of clusters and the best suitable values of penalty parameters, usually multiple values of $K$, $\lambda_{1}^{(c_{i})}$ and $\lambda_{2}^{(c_{i})}$are set and the best model is selected via comparing the EBIC scores [4] among results.

**References**

[1] Liu DC, Nocedal J. On the limited memory BFGS method for large scale optimization. Math.program.b 1989;45:503-28.

[2] Hsieh CJ, Dhillon IS, Ravikumar P. QUIC: quadratic approximation for sparse inverse covariance estimation. Journal of Machine Learning Research 2014;15:2911-47.

[3] Zhong K, Yen IEH, Dhillon IS, Ravikumar P. Proximal Quasi-Newton for Computationally Intensive L1-regularized M-estimators. Advances in Neural Information Processing Systems 2014;3:2375-83.

[4] Chen J, Chen Z. Extended Bayesian Information Criteria for Model Selection with Large Model Spaces. Biometrika 2008;95:759-71.
