# Supplementary figures and images for: kLDM: Inferring Multiple Metagenomic Association Networks Based on the Variation of Environmental Factors
Source: Genomics Proteomics Bioinformatics. 2021 Feb 17;19(5):834–47. doi: 10.1016/j.gpb.2020.06.015 (PMC9170748; doi:10.1016/j.gpb.2020.06.015)

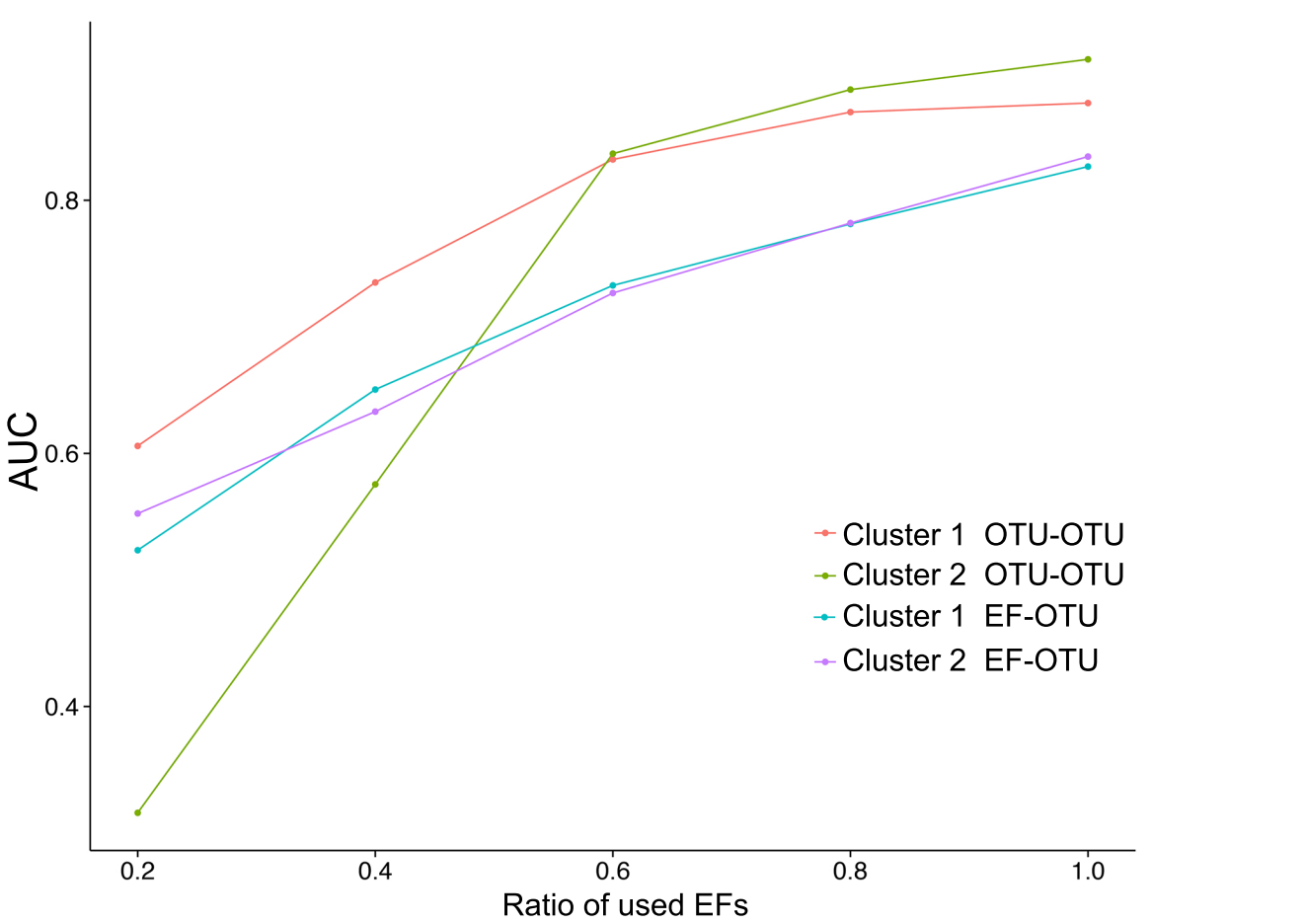

Supplement: Supplementary Figure S1 — AUC scores of kLDM when EFs with different ratios are utilized The ‘baseline’ dataset in Table S3 was used, and the results when only 20%, 40%, 60%, and 80% of the EFs found in the dataset were compared. EFs were orderly selected and set to zero because they equally contributed to EF-OTU associations in the simulation experiment. The changes of the AUCs of the OTU-OTU and EF-OTU associations of two clusters are shown. [file mmc2.pdf]

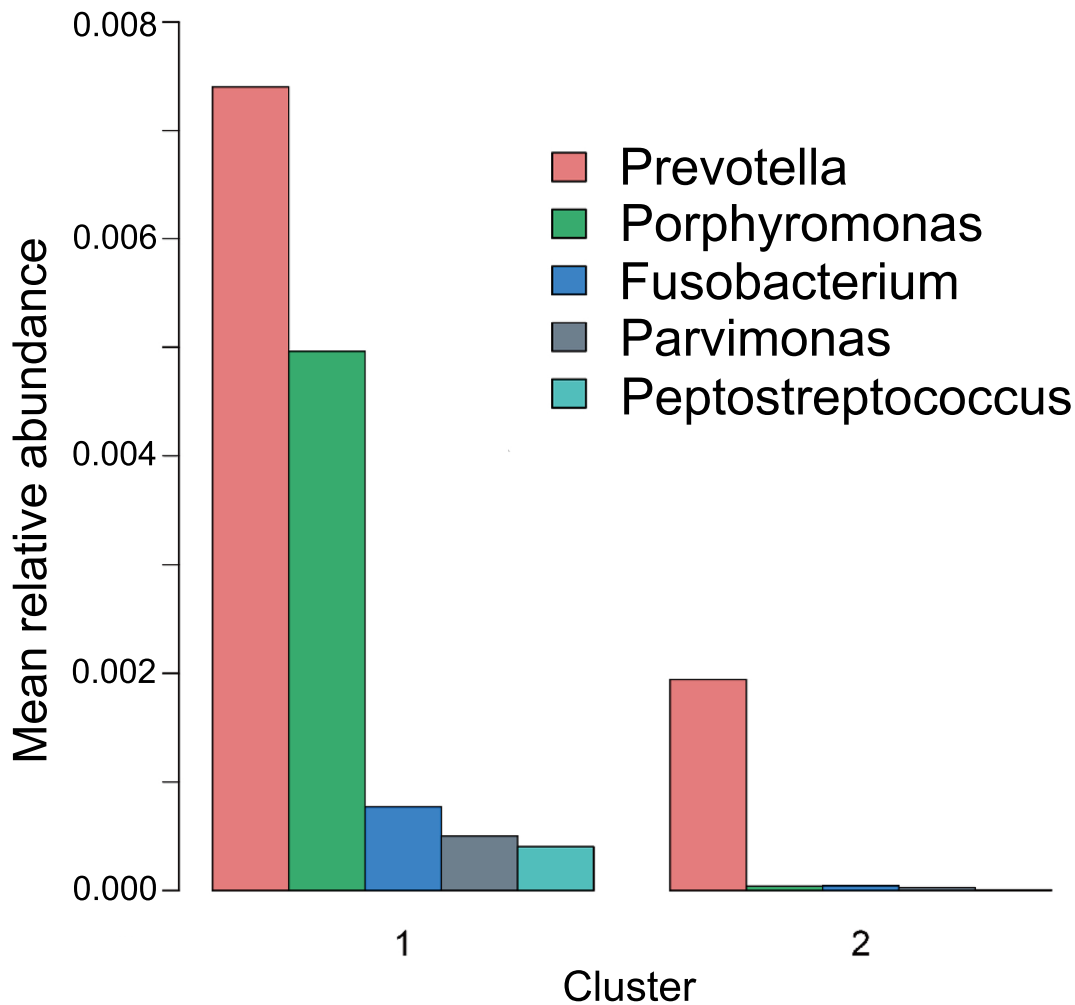

Supplement: Supplementary Figure S2 — CRC-associated microbial relative abundances of two clusters [file mmc3.pdf]

A OTU-OTU associations of Cluster 1

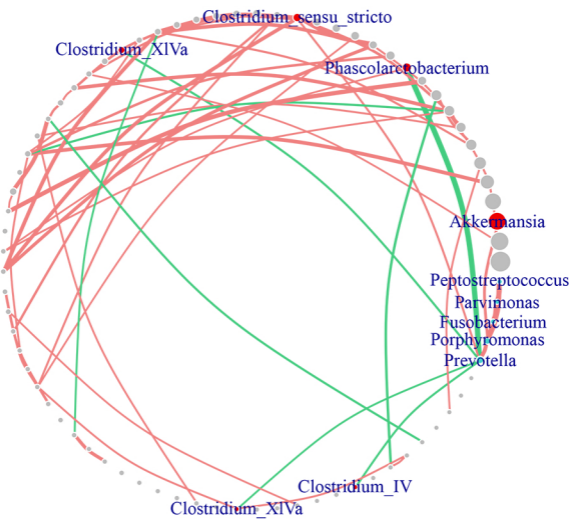

B OTU-OTU associations of Cluster 2

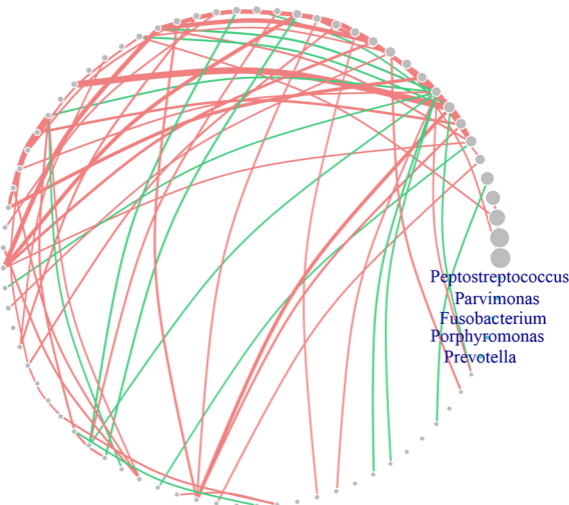

Supplement: Supplementary Figure S3 — OTU-OTU association networks for cancer (Cluster 1) and healthy (Cluster 2) clusters Edges represent associations with absolute weight among the top 1%. Red and green edges represent positive and negative associations, respectively. The width of an edge is proportional to the association’s absolute weight. Only microbes with associations in either Cluster 1 or Cluster 2 are shown. The positions of OTUs in A and B are exactly identical, and the sizes of nodes are correlated with their average abundance in all samples. 5 CRC-associated microbes (Peptostreptococcus, Parvimonas, Fusobacterium, Porphyromonas, and Prevotella) are arranged closely and colored in blue-green, and their associated microbes are labeled in red. [file mmc4.pdf]

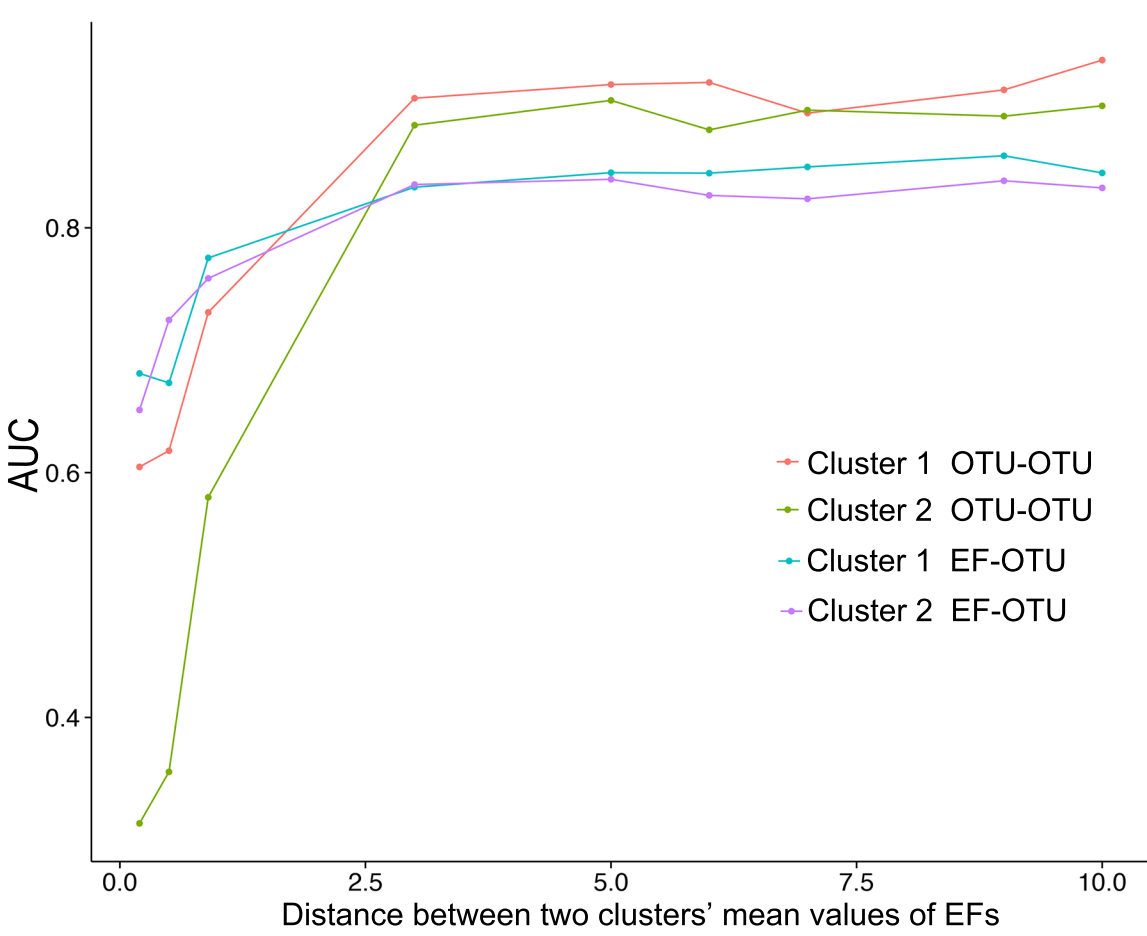

Supplement: Supplementary Figure S4 — The relationship between the effectiveness of kLDM and the similarity of the EFs of EF conditions The ‘baseline’ dataset in Table S3 was used again, and the distances between the mean values of the EFs of the two clusters were changed from 0.1 to 10.0. For each distance, AUC scores of the estimated OTU-OTU associations (“Cluster 1 OTU-OUT” and “Cluster 2 OTU-OUT”) and EF-OTU associations (“Cluster 1 EF-OUT” and “Cluster 2 EF-OUT”) of the two clusters were calculated. [file mmc5.pdf]
