# Supplementary material for: kLDM: Inferring Multiple Metagenomic Association Networks Based on the Variation of Environmental Factors
Source: Genomics Proteomics Bioinformatics. 2021 Feb 17;19(5):834–47. doi: 10.1016/j.gpb.2020.06.015 (PMC9170748; doi:10.1016/j.gpb.2020.06.015)
Supplement: Supplementary Table S1 — Comparison of the running time and memory usage of kLDM and mLDM on a single association network inference [file mmc6.docx]

## Table S1 Comparison of running time and memory usage by kLDM and mLDM on single association network inference

| **Dataset (hub graph)** | **kLDM (4 cores)** | | **kLDM (8 cores)** | | **mLDM (Rcpp)** | | **mLDM (R language)** | |
| --- | --- | --- | --- | --- | --- | --- | --- | --- |
|  | **Time** | **Memory** | **Time** | **Memory** | **Time** | **Memory** | **Time** | **Memory** |
| P = 50, Q = 5, N = 500 | 10 min 51 s | 192 MB | 5 min 49 s | 175 MB | 23 min 19 s | 226 MB | 1 h 13 min | 300 MB |

*Note:* A synthetic dataset with 50 OTUs, 5 EFs, and 500 samples was constructed and all programs were run on a server with CentOS 7.4 operating system and Intel(R) Xeon(R) E5-2680 v3 @ 2.50GHz CPUs. The ‘mLDM (R language)’ is implemented in R language, the ‘mLDM (Rcpp)’ rewrites original R functions in C++ language and integrates codes into a R package. In kLDM, we use the pure C++ codes to infer association networks and add the ability of multithread processing. OTU, operational taxonomic unit; EF, environmental factor; kLDM, k-Lognormal-Dirichlet-Multinomial model; mLDM, metagenomic Lognormal-Dirichlet-Multinomial model; Rcpp, R and C++ integration.
