# Supplementary material for: kLDM: Inferring Multiple Metagenomic Association Networks Based on the Variation of Environmental Factors
Source: Genomics Proteomics Bioinformatics. 2021 Feb 17;19(5):834–47. doi: 10.1016/j.gpb.2020.06.015 (PMC9170748; doi:10.1016/j.gpb.2020.06.015)
Supplement: Supplementary Table S4 — AUC scores of kLDM when EFs with different ratios are utilized and other methods use the full dataset [file mmc9.docx]

## Table S4 AUC scores of kLDM when EFs with different ratios are utilized and other methods with full dataset

| **Method** | **Cluster1 OTU-OTU** | **Cluster1 EF-OTU** | **Cluster2 OTU-OTU** | **Cluster2 EF-OTU** |
| --- | --- | --- | --- | --- |
| **SCC (all)** | 0.69 $\pm$0.02 | 0.52 $\pm$ 0.02 | 0.50 $\pm$ 0.00 | 0.45 $\pm$ 0.02 |
| **CCLasso** | 0.59 $\pm$ 0.04 | - | 0.70 $\pm$ 0.02 | - |
| **SPIEC** | 0.50 $\pm$ 0.00 | - | 0.50 $\pm$ 0.00 | - |
| **SCC** | 0.55 $\pm$ 0.05 | 0.76 $\pm$ 0.09 | 0.55 $\pm$ 0.01 | 0.73 $\pm$ 0.03 |
| **kLDM (100%)** | 0.88 $\pm$ 0.11 | 0.83 $\pm$ 0.08 | 0.91 $\pm$ 0.04 | 0.83 $\pm$ 0.03 |
| **EF (80%)** | 0.86 $\pm$ 0.11 | 0.78 $\pm$ 0.04 | 0.88 $\pm$ 0.08 | 0.78 $\pm$ 0.03 |
| **EF (60%)** | **0.83** $\pm$ **0.11** | **0.73** $\pm$ **0.05** | **0.84** $\pm$ **0.08** | **0.72** $\pm$ **0.05** |
| **EF (40%)** | 0.74 $\pm$ 0.10 | 0.65 $\pm$ 0.07 | 0.58 $\pm$ 0.20 | 0.63 $\pm$ 0.04 |
| **EF (20%)** | 0.61 $\pm$ 0.03 | 0.52 $\pm$ 0.04 | 0.32 $\pm$ 0.08 | 0.55 $\pm$ 0.03 |

*Note:* Results of kLDM matched with the **Figure S5** and four other methods’ results on the whole dataset are listed. The ‘EF (20%)’ means 20% EFs are employed by kLDM to infer association networks. The AUC score is formatted with 'mean value $\pm$ standard deviation'. OTU-OTU, microbe-microbe; EF, environmental factor; EF-OTU, environmental factor-microbe; kLDM, k-Lognormal-Dirichlet-Multinomial model; SCC, spearman correlation coefficient.
