# Supplementary material for: kLDM: Inferring Multiple Metagenomic Association Networks Based on the Variation of Environmental Factors
Source: Genomics Proteomics Bioinformatics. 2021 Feb 17;19(5):834–47. doi: 10.1016/j.gpb.2020.06.015 (PMC9170748; doi:10.1016/j.gpb.2020.06.015)
Supplement: Supplementary Table S5 — Mean values of the metadata of two clusters on colorectal cancer data [file mmc10.docx]

## Table S5 Mean values of meta data of two clusters on colorectal cancer data

| **Meta Name** | **Cluster 1** | **Cluster 2** | **P-value** |
| --- | --- | --- | --- |
| FIT | 531.15 | 8.47e-7 | 7.18e-22 |
| Age | 61.81 | 59.05 | 0.013 |
| Gender (Female) | 46.89% | 53.31% | 0.066 |

*Note:* Mean values of FIT, age and gender are calculated and single factor two-tailed T-test is used to decide significance. FIT, fecal immunochemical test results.
