# Supplementary material for: kLDM: Inferring Multiple Metagenomic Association Networks Based on the Variation of Environmental Factors
Source: Genomics Proteomics Bioinformatics. 2021 Feb 17;19(5):834–47. doi: 10.1016/j.gpb.2020.06.015 (PMC9170748; doi:10.1016/j.gpb.2020.06.015)
Supplement: Supplementary Table S6 — Compositions of the diagnostic states in two clusters on colorectal cancer data [file mmc11.docx]

## Table S6 Composition of the diagnostic state in two clusters on colorectal cancer data

| Name | Normal | High Risk Normal | Adenoma | Advanced Adenoma | Cancer |
| --- | --- | --- | --- | --- | --- |
| Cluster 1 | 19 | 9 | 48 | 34 | **108** |
| Cluster 2 | **103** | 41 | 61 | 55 | 12 |

*Note:* The number of samples with corresponding diagnostic state in two clusters is listed.
