# Supplementary material for: kLDM: Inferring Multiple Metagenomic Association Networks Based on the Variation of Environmental Factors
Source: Genomics Proteomics Bioinformatics. 2021 Feb 17;19(5):834–47. doi: 10.1016/j.gpb.2020.06.015 (PMC9170748; doi:10.1016/j.gpb.2020.06.015)
Supplement: Supplementary Table S8 — Matched genus-level interactions on the Tara Oceans dataset by kLDM and “Static” [file mmc13.docx]

## Table S8 Matched genus-level interactions on TARA Oceans dataset of kLDM and the 'Static'

| Method | kLDM Cluster1 | kLDM Cluster2 | 'Static' |
| --- | --- | --- | --- |
| MG @ Top 10 | 2 | 0 | 2 |
| MG @ Top 20 | 2 | 2 | 4 |
| MG @ Top 40 | 5 | - | 6 |
| MG @ Top 60 | 7 | - | 8 |
| MG @ Top 80 | 8 | - | 9 |
| MG @ Top 100 | 9 | - | 13 |
| MG @ Top 120 | 13 | - | 15 |

*Note:* ‘MG @ Top N’ represents matched known genus-level interactions' number among top N predicted associations. the flag ‘-’ corresponds the entry where the number of predictions is < N. Results of two clusters of kLDM are listed separately. The 'Static' was estimated by assuming that there was only one cluster in the dataset. MG, matched known genus-level interactions; kLDM, k-Lognormal-Dirichlet-Multinomial model.
