# Supplementary material for: kLDM: Inferring Multiple Metagenomic Association Networks Based on the Variation of Environmental Factors
Source: Genomics Proteomics Bioinformatics. 2021 Feb 17;19(5):834–47. doi: 10.1016/j.gpb.2020.06.015 (PMC9170748; doi:10.1016/j.gpb.2020.06.015)
Supplement: Supplementary Table S15 — Values of the inverse Simpson index of synthetic datasets corresponding to Figure 2 and 3 [file mmc20.docx]

**Table S15 Values of inverse Simpson index of synthetic datasets corresponding to Figure 2 and Figure S1**

| Synthetic datasets | Inverse Simpson index $n_{eff}$ |
| --- | --- |
| K = 2, P = 50, Q = 5 and N ∈ [100,200] | 9.52 |
| K = 2, P = 50, Q = 5 and N ∈ [200,400] | 9.48 |
| K = 2, P = 100, Q = 8 and N ∈ [400,800] | 16.82 |
| K = 2, P = 200, Q = 10 and N ∈ [800,1600] | 33.22 |

*Note:* The inverse Simpson index can be calculated by $n_{eff}=e^{-\sum_{j=1}^{P} x_{j}logx_{j}}$ where $x_{j}$ is the relative abundance of the $j^{th}$ OTU.
