# Supplementary material for: kLDM: Inferring Multiple Metagenomic Association Networks Based on the Variation of Environmental Factors
Source: Genomics Proteomics Bioinformatics. 2021 Feb 17;19(5):834–47. doi: 10.1016/j.gpb.2020.06.015 (PMC9170748; doi:10.1016/j.gpb.2020.06.015)
Supplement: Supplementary Table S16 — Compositions of the diagnostic states in two clusters estimated by MicrobeDMM on colorectal cancer data [file mmc21.docx]

## Table S16 Composition of the diagnostic state in two clusters estimated by MicrobeDMM on colorectal cancer data

| Name | Normal | High Risk Normal | Adenoma | Advanced Adenoma | Cancer |
| --- | --- | --- | --- | --- | --- |
| Cluster 1 | 50 | 10 | 43 | 17 | 54 |
| Cluster 2 | 72 | 40 | 66 | 72 | 66 |

*Note:* The number of samples with corresponding diagnostic state in two clusters is listed. MicrobeDMM, microbe dirichlet multinomial model.
