# Supplementary material for: kLDM: Inferring Multiple Metagenomic Association Networks Based on the Variation of Environmental Factors
Source: Genomics Proteomics Bioinformatics. 2021 Feb 17;19(5):834–47. doi: 10.1016/j.gpb.2020.06.015 (PMC9170748; doi:10.1016/j.gpb.2020.06.015)
Supplement: Supplementary Table S17 — IBD diagnosis of IBD patients in three clusters on the American Gut Project dataset [file mmc22.docx]

## Table S17 IBD diagnosis of IBD patients in three clusters on the American Gut project dataset

| Disease Name | IBD in C1 | IBD in C2 | IBD In C3 |
| --- | --- | --- | --- |
| Colonic Crohn's disease | 7 | 1 | **106** |
| Ileal and colonic Crohn's disease | 5 | 1 | 0 |
| Ileal Crohn's disease | 11 | 6 | 0 |
| Microcolitis | 3 | 0 | 0 |
| ulcerative colitis | 35 | 18 | 0 |
| Unknown | **163** | **40** | 0 |
| SUM | 224 | 66 | 106 |

*Note:* C1, C2, and C3 are three clusters estimated by kLDM. IBD, inflammatory bowel disease.
